# High costs, low quality of life, reduced survival, and room for improving treatment: an analysis of burden and unmet needs in glioma

**DOI:** 10.3389/fonc.2024.1368606

**Published:** 2024-03-20

**Authors:** Johannes Pöhlmann, Michael Weller, Andrea Marcellusi, Kristin Grabe-Heyne, Lucia Krott-Coi, Silvia Rabar, Richard F. Pollock

**Affiliations:** ^1^ Covalence Research Ltd, Harpenden, United Kingdom; ^2^ Department of Neurology, University Hospital and University of Zurich, Zurich, Switzerland; ^3^ Economic Evaluation and HTA (EEHTA)-Centre for Economic and International Studies (CEIS), Faculty of Economics, University of Rome “Tor Vergata”, Rome, Italy; ^4^ Medac GmbH, Wedel, Germany

**Keywords:** cost, glioblastoma, glioma, lomustine, quality of life, treatment pattern, unmet need

## Abstract

Gliomas are a group of heterogeneous tumors that account for substantial morbidity, mortality, and costs to patients and healthcare systems globally. Survival varies considerably by grade, histology, biomarkers, and genetic alterations such as IDH mutations and *MGMT* promoter methylation, and treatment, but is poor for some grades and histologies, with many patients with glioblastoma surviving less than a year from diagnosis. The present review provides an introduction to glioma, including its classification, epidemiology, economic and humanistic burden, as well as treatment options. Another focus is on treatment recommendations for IDH-mutant astrocytoma, IDH-mutant oligodendroglioma, and glioblastoma, which were synthesized from recent guidelines. While recommendations are nuanced and reflect the complexity of the disease, maximum safe resection is typically the first step in treatment, followed by radiotherapy and/or chemotherapy using temozolomide or procarbazine, lomustine, and vincristine. Immunotherapies and targeted therapies currently have only a limited role due to disappointing clinical trial results, including in recurrent glioblastoma, for which the nitrosourea lomustine remains the *de facto* standard of care. The lack of treatment options is compounded by frequently suboptimal clinical practice, in which patients do not receive adequate therapy after resection, including delayed, shortened, or discontinued radiotherapy and chemotherapy courses due to treatment side effects. These unmet needs will require significant efforts to address, including a continued search for novel treatment options, increased awareness of clinical guidelines, improved toxicity management for chemotherapy, and the generation of additional and more robust clinical and health economic evidence.

## 1 Introduction

Malignant brain tumors are associated with a morbidity, mortality, and economic burden that is substantial not only in absolute terms but also relative to other cancers, despite the comparatively smaller number of patients with these tumors (1–3). Most malignant brain tumors (approximately 80%) are gliomas (4). Gliomas arise from neuroglial progenitor cells in the brain or spinal cord and form a heterogeneous group of tumors differentiated by histology, location, and anaplastic features (3). The most prevalent and aggressive type of glioma is glioblastoma, which accounts for half of all malignant brain tumors (5). Survival in patients with glioblastoma remains exceptionally poor, with a 5-year survival of 2–10% (5, 6).

The present review provides an introduction to adult-type glioma and an overview of its epidemiology, before characterizing its humanistic and economic burden. In addition, existing treatment options and guidelines for glioma are reviewed, with a focus on the use of the cytostatic nitrosourea derivative lomustine across treatment lines. The review and its focus on treatment (screening, testing, diagnosis, and palliative care are not covered here) are expected to be useful to a wide audience, including clinicians new to the field and to non-clinical researchers working in health economics, health technology assessment, and pricing/reimbursement agencies.

## 2 Disease classification, grading, and associated survival

### 2.1 The WHO 2021 classification of glioma

Gliomas are classified based on histology and molecular biomarkers (7). Among the molecular biomarkers, isocitrate dehydrogenase (IDH) mutations and chromosome 1p/19q codeletion are key for defining glioma types and subtypes, but a wide range of additional genetic and molecular alterations (e.g., in H3 K27 and G34) are also known to be relevant for disease classification.

The 2021 WHO classification of central nervous system (CNS) tumors (8) distinguished between six types of gliomas, glioneuronal tumors, and neuronal tumors, with this review focusing on adult-type diffuse gliomas and its three subtypes. The 2021 WHO classification substantially revised the preceding classification from 2016. Changes included simplifying tumor names, removing modifier terms such as “anaplastic”, distinguishing adult- and pediatric-type gliomas, and limiting the diagnosis of glioblastoma to IDH-wildtype disease. The previously defined variant of IDH-mutated glioblastoma was reclassified as astrocytoma, IDH-mutant, CNS WHO grade 4.

These and further revisions were made to reflect advances in the field, most notably around the molecular understanding of gliomas, and to allow selecting more specific therapies (7, 9). Concerns have been raised, however, about the feasibility of implementing molecular testing at the required scale and with clinically appropriate turnaround times, especially given the limited availability of treatments for specific tumors (9). A fine-grained distinction based on molecular markers might also decrease the sample sizes available to clinical studies, thereby increasing the challenges of patient recruitment (9). It is therefore likely advisable to consider, as suggested by Louis et al. (7), even the latest classification as a work in progress that can be refined if and when new evidence becomes available.

### 2.2 Glioma survival in relation to type/variant and grade

Glioma type and entity (the term used in the WHO 2016 classification now changed to “type”) are associated with patient survival (**Supplementary Table S1** in the Electronic **Supplementary Material** [ESM]) (5, 10–27). Survival is poorest in patients with glioblastoma, where median overall survival (OS) was between 9 (11) and 26 (14) months. Survival at 1 year varied between 29% (17) and 54% (19), survival at 2 years between 10% (26) and 27% (26). Age-standardized 5-year net survival was estimated to range between 4% and 17% (15), with some evidence for broadly improving trends globally, while 5-year OS ranged between 2% (25) and 10% (18).

#### 2.2.1 Glioblastoma

In glioblastoma, progression predicts shorter OS as shown in recent analyses from the ETERNITY, where the median OS in patients surviving ≥5 years from glioblastoma diagnosis was 9.9 years overall but was not reached in patients without recurrence relative to 8.9 years in patients with ≥1 recurrence (16). Unsurprisingly, survival in glioblastoma is associated with treatment, including surgery and chemotherapy; in Norway, median OS was reported as 6.8 months in those receiving only biopsy and 17.2 months in those with gross total resection (10). Similar data were available for the UK, where biopsy only was associated with a median OS of 8.0 months relative to 14.9 months in those with debulking surgery (11), and for Denmark, where biopsy only was associated with a median OS of 4.7 months, compared with 15.6 months in those with gross total resection (28). Patients with glioblastoma in France who received no oncologic treatment had a median OS of only 1.8 months, compared with 16.4 and 18.9 months in those receiving radiotherapy and concomitant or adjuvant temozolomide (TMZ), respectively, while median OS extended to 26 months in those receiving radiotherapy and ≥6 cycles of TMZ (14).

#### 2.2.2 Astrocytoma

In astrocytoma, median OS was reported to vary between 5.8 months in elderly patients (20) to 23.8 months in diffuse grade 4, *IDH1/2* wildtype astrocytoma with molecular features of glioblastoma (22) and to 5.2 years for low-grade astrocytoma (12). At 1 year, OS varied considerably by grade for IDH-mutant astrocytoma, from 76% in grade 4 to 98% in grade 2 (19), but was also reported as only 48% in Canadian patients with anaplastic astrocytoma (26). Age-standardized 5-year net survival for diffuse and anaplastic astrocytoma globally was estimated at 20% to 38%, with positive or at least stable trends (15), while 5-year OS ranged from 11% for anaplastic astrocytoma (25) to 75% for diffuse astrocytoma (18) and 91% for any astrocytoma (27).

#### 2.2.3 Oligodendroglioma

Compared with glioblastoma and astrocytoma, oligodendroglioma is associated with better survival. Median OS ranged from 22.6 months in elderly patients (20) to 4.6 years in anaplastic oligodendroglioma treated with procarbazine, lomustine, and vincristine (PCV) and radiotherapy (29) and to 7.2 years in grade 2 disease (12). The age-adjusted 5-year net survival was estimated at 32–69%, with generally improving trends (15). Five-year OS for anaplastic oligodendroglioma ranged between 35% (25) to 94% and 98% in IDH-mutant, 1p/19q codeleted oligodendroglioma with grade 2 and grade 3, respectively (19).

## 3 Epidemiology

The age-standardized incidence rate (ASIR) of glioma, regardless of histology, was reported to vary between 4.67 [in Finland (30)] and 7.1 [in the United Kingdom [UK] (31)] cases per 100,000 population (**Supplementary Table S2** in the ESM). Gliomas occur more often in men than in women, and their incidence increases with age (as does the incidence ratio for men versus women). Except for the youngest patients (aged 0–9 years), in whom female patients have a higher risk of death than male patients, men are also at an increased risk of glioma mortality compared with women (32). Data for glioma mortality are sparse, particularly when compared with survival data, but recent data from the United States (US) suggested an overall age-standardized mortality rate of 4.33 deaths per 100,000 population (5).

### 3.1 Glioblastoma

Glioblastoma was, by far, the most frequently occurring type of glioma, but reported ASIRs displayed substantial variation across country settings. Some of the lowest ASIRs were reported for the US, with 3.20 (95% confidence interval [CI] 3.17 to 3.23) cases per 100,000 population (5) Australia, with 3.96 cases per 100,000 population (33), and for Spain, with 4.17 (95% CI 3.80 to 4.57) cases per 100,000 population (34). Higher rates were reported for France, with 5.3 cases per 100,000 population (35), and the UK, with 7.1 (95% CI: 6.5 to 7.8) cases per 100,000 population (31).

Glioblastoma ASIRs in men are 50–60% higher than in women, across age groups and ethnicities (5, 32, 34–38). The incidence of glioblastoma rises steadily with age before plateauing in people aged ≥65 years (5, 32), reaching up to 13.2 (95% CI: 13.0 to 13.3) cases per 100,000 population among elderly people in the US (36). Significant increases in glioblastoma incidence have been observed in several countries, including Australia, with an annual percentage change [APC] of 2.5% between 2000 and 2008 (33), and the UK, with increases in ASIRs per 100,000 population between 1995 and 2017 from 3.27 to 7.34 cases in men and 2.00 to 4.45 cases in women (37).

### 3.2 Astrocytoma and oligodendroglioma

The incidence of astrocytoma and oligodendroglioma is lower by approximately an order of magnitude compared with glioblastoma (**Supplementary Table S2** in the ESM) (5, 34, 35). Based on the WHO 2016 classification, which was used in most currently available studies, the incidence is highest for diffuse astrocytoma, ranging between 0.23 (35) and 0.93 (34) cases per 100,000 population, followed by anaplastic (0.31 (35) and 0.48 (34) per 100,000 population) and pilocytic astrocytoma (0.19 (35) to 0.36 (5) per 100,000 population). The incidence of oligodendroglioma is lower again, ranging between 0.15 (35) and 0.24 (5) cases per 100,000 population for oligodendroglioma in general and 0.05 (34) to 0.11 (5) per 100,000 population for anaplastic oligodendroglioma.

Unlike for glioblastoma, there is no consistent pattern regarding the association of incidence with sex. While anaplastic astrocytoma and oligodendroglioma were reported to occur more frequently in men than in women in Spain (34), US data indicated that pilocytic astrocytoma occurred at similar frequency in each sex while other histologies were more frequent in men than in women (5). In France, in contrast, anaplastic oligodendroglioma was more frequent in women than men, with similar incidences for pilocytic astrocytoma (35). These data suggested country-specific incidence profiles for non-glioblastoma histologies, but frequently small numbers of patients and subsequent uncertainty in the estimates should be acknowledged for these histologies.

## 4 Burden

### 4.1 Economic burden

#### 4.1.1 Direct costs of glioma to healthcare systems

##### 4.1.1.1 US

In the US, to which most of the economic literature on glioma pertains, the disease is associated with a substantial resource and economic burden, although cost estimates vary over time, by line of therapy, and by type of insurance (**Table 1**).

**Table 1:** Overview of economic and humanistic burden associate with glioma.

Item	Summary of findings
*Economic burden*	
United States	• Mean estimates of cumulative per-patient costs range from USD 95,377 (Medicare; driven mainly by hospitalization) for glioblastoma (39) to USD 184,160–268,031 (commercial, driven by in- and outpatient costs) across high-grade gliomas (40, 41) • Patients with brain cancer (not just glioma) face financial hardship, with >10% of patients or caregivers reporting delayed or unaffordable care (42)
Globally	A 2021 review identified wide variation between countries and institutions (as well as poor reporting) of glioblastoma-associated management costs (43): mean direct costs per patient for surgical resection, in 2017 USD, were USD 10,042 (ranging from USD 4,128 to USD 14,857), with combined surgery, chemotherapy, and radiotherapy estimated at USD 62,602
Productivity losses, indirect costs	• Productivity losses – due to the disease, associated depression, and the inability to return to work – account for a substantial cost burden in glioma • In the Netherlands, glioma-associated productivity losses per 4 weeks were EUR 1,265 (patients) and EUR 337 (caregivers), due to health problems, absenteeism, and presenteeism in 2015 (44) • In Spain, overall indirect costs per patient with glioblastoma were EUR 111,926 in 2015 (45) • Economic losses due to premature death from brain cancer in Europe (2018) were EUR 428,449 per premature death, due to lost paid production (1)
*Humanistic burden*	
Quality of life with glioma	• Generally, quality of life is reduced with glioma, mainly due to disease symptoms and signs, which include neurocognitive deficits (such as visual disorders and problems communicating), disturbed sleep, fatigue, drowsiness, reduced bladder control, and itchy skin (46–51) • For up to 81% of patients, quality of life is the most important factor in treatment decisions, and 79% value quality of life over survival at diagnosis (52)
Change in quality of life	• Quality of life tends to remain stable after surgery or improves slightly in the long term, including in low-grade glioma (49, 53) and glioblastoma (54) • Advanced/high-grade gliomas more likely lead to worse quality of life and mood disorders than low-grade gliomas, they are also more likely to be associated with deteriorating quality of life while quality of life in lower-grade gliomas often remains stable (55–57)
Treatment-related quality of life	• No significant quality of life differences when comparing lomustine with regorafenib (58) or as an add-on to temozolomide (59) • No significant quality of life difference between patients with low-grade glioma receiving radiotherapy or temozolomide (60)

The mean first-line treatment costs for diffuse low-grade glioma for the 90 days following supratentorial resection and stereotactic biopsy were at USD 56,093 (standard deviation [SD] 67,974) and USD 43,219 (SD 65,463), respectively, in 2014 USD (61). These costs were driven by the costs of the index procedures, estimated at USD 39,043 (SD 44,391) and USD 40,661 (SD 47,068), respectively. The mean costs of chemotherapy drugs, over the 90 days after the operation, were estimated at USD 12,717 (SD 6,782) and USD 12,752 (SD 6,976). For high-grade glioma, the median (95% CI) healthcare payments amounted to USD 184,160 (151,215 to 222,431; cost year not reported) (40). Out- and inpatient payments accounted for 35% and 25% of costs, respectively, compared with only 12% for prescription drugs. Over time, costs were highest in the initial treatment (USD 66,673) and the recurrence phase (USD 52,126), while the maintenance phase (defined as the period after surgery or (chemo-) radiation therapy, while receiving TMZ or no chemotherapy) was associated with comparatively low costs (USD 14,491).

Costs are even higher for glioblastoma. Newly diagnosed glioblastoma was associated, in Medicare patients (aged ≥66 years), with cumulative mean per-patient costs of USD 95,377 and per-patient per-month (PPPM) costs of USD 18,053 (cost year not reported), over a median OS of only 5.9 months (39). These costs were driven by hospital (inpatient) costs and the costs of surgical resection. Nearly all (90%) patients were admitted to an emergency room after their diagnosis, and 86% were admitted to an ICU. First-line systemic therapy was associated with PPPM costs of USD 124,138 in the post-diagnosis period, considerably more than in patients only receiving radiotherapy (USD 79,009). Estimates reported by Norden et al. (62) for private insurance and Medicare beneficiaries confirmed these high costs in glioblastoma. Mean total per patient costs after initiating first-line therapy were USD 117,325 at 6 months and USD 162,550 at 12 months, in 2016 USD. Radiotherapy and systemic therapy drove first-line treatment costs, while systemic therapy was the main driver of second-line therapy costs, which were estimated at USD 126,128 at 6 months and USD 243,833 at 12 months (62).

Commercial data yielded similar results. In newly diagnosed, TMZ-treated patients with glioblastoma, a median of two inpatient, one emergency department, and nineteen outpatient visits took place after diagnosis, with a median length of stay per hospitalization of 5 days (41). The combined resource use translated to mean cumulative per-patient costs between 3 months before and 12 and 60 months after diagnosis, respectively, of USD 201,749 and USD 268,031 (cost year not reported but likely 2014) (41).

##### 4.1.1.2 Countries besides the US

Cost estimates for countries besides the US were available from a systematic review by Goel et al. (43), which included twenty-one studies measuring direct costs related to glioblastoma management in Europe, North America and China (**Table 1**). Direct costs for surgical resection varied from USD 4,128 to USD 14,857, with a mean of USD 10,042 (2017 USD), while the mean radiotherapy cost was USD 6,777. Combining surgery, radiation, and chemotherapy was associated with an OS of 16.3 months and a mean cost of USD 62,602. Importantly, the authors deduced that estimates varied widely, between countries and between institutions within countries, and often fell short of being comprehensive. Standards of care, recurrence costs, and incremental costs associated with specific therapies were therefore challenging to compare (43).

#### 4.1.2 Costs of glioma to patients and productivity losses due to glioma

Glioma is associated with noteworthy costs to patients and indirect costs, mostly due to productivity losses (**Table 1**).

##### 4.1.2.1 United States

In the US, patients bear part of the direct medical costs out-of-pocket. In patients with low-grade glioma, patients paid USD 811–1,164 out of pocket for drugs and index procedures in the first 90 days after surgery (61). The out-of-pocket costs for chemotherapy amounted to USD 274–301, in addition to USD 40–56 for anti-epileptic drugs. These and similar expenses may be difficult to cover for patients, as shown in a cross-sectional study by Desai et al. (42), who investigated the financial hardship of patients with brain tumors in the USA between 1997 and 2018. Over 10% of the respondents delayed or avoided medical care in the past year because of costs. Regarding insurance status, uninsured patients had the highest prevalence of affirmative responses to all four questions, and the difference between the insurance coverage subgroups was significant (42).

Even stable, high-functioning survivors of low- and high-grade glioma experience a substantial financial burden (including high personal debt) and workforce morbidity (63). Almost one in four patients have been shown to incur brain tumor-related debts, more than half required unpaid time off work, and 46% retired or stopped working. These patterns suggested high indirect costs and productivity losses across glioma subtypes, treatment lines and income groups (63). Returning to work was a particular concern, especially among lower-grade glioma survivors, who were often younger and working at the time of their glioma diagnosis (64). On average, rates of return to work identified in a systematic review indicated that 73% of patients returned to work (range: 31–97%), after a mean 6.3 months (range: 15 days to 22 months). Among treatment factors influencing return to work, improved neurologic status, larger resection extent, and absence of seizures were associated with higher rates of return to work (64).

##### 4.1.2.2 Europe

Productivity losses associated with glioma have also been quantified for European countries. Boele et al. (44) assessed the impact of psychological symptoms on glioma patients and their caregivers. Data were taken from a Dutch randomized controlled trial (RCT) conducted between 2011 and 2015. Yearly overall (direct and indirect) costs were EUR 20,857.53 for patients and EUR 5,581.49 for caregivers. Costs for productivity loss per 4 weeks were EUR 1264.95 per patient (employed and unemployed) and EUR 337.42 per caregiver. Costs varied between participants. While only one third of patients and two thirds of caregivers were in paid employment, productivity losses accounted for over 78% of costs. Of note, most costs were divided evenly across the Dutch population through the social security system, and thus reflected a societal rather than personal cost (44).

At work, people with glioma face unique challenges, such as difficulties in reintegrating into the workplace (including physicians, colleagues, and supervisors who fail to understand the wish to return to work), concerns by line managers and colleagues regarding dependability, and a lack of tailored support structures at work (65).

The productivity losses due to premature death were estimated, for Europe, at EUR 428,449 per premature death due to brain cancer (**Table 1**) (1). These losses were mostly due to lost paid production in employed people, and ranked among the highest across cancer sites.

### 4.2 Humanistic burden

Glioma is associated with impaired health-related quality of life (HRQoL) in both patients and their caregivers, and while HRQoL is often stable over time, it consistently remains below general population norms in long-term glioma survivors. The currently available evidence, however, does not suggest differential HRQoL impacts for different treatment options.

#### 4.2.1 Burden of disease

##### 4.2.1.1 Patients

Glioma affects patients’ functional status and HRQoL negatively, due to disease- and therapy-related symptoms such as neurocognitive deficits, including visual disorders and communication deficits, disturbed sleep, fatigue, drowsiness, itchy skin, and bladder control issues, and due to associated conditions such as depression and anxiety (**Table 1**) (3, 46–49, 66). Concerns about HRQoL are the main factor in treatment decisions for up to 81% of patients, of whom 81% and 79% – for low- and high-grade glioma, respectively – are primarily concerned with quality of life, not survival, when deciding on treatment (52). Assessing patient-reported outcomes (PROs) in routine care is favored by patients, caregivers, and healthcare providers (67).

###### 4.2.1.1.1 Drivers of reduced HRQoL

Neurological deficits impact 44–50% of patients with glioma before surgery (52, 68) and are more than three times more frequent in patients with diffuse glioma than in age- and sex-matched people from the general population (68), with cognitive performance often declining over time (69). These deficits have been identified as reducing HRQoL, with new-onset deficits associated with a reduction of 0.17 in EQ-5D 3L index values relative to no deficit in diffuse lower-grade glioma (46). Data by histology suggested that patients with glioblastoma had worse HRQoL and functioning on domains such as emotion than patients with other glioma (and brain tumors generally) (48).

Disease recurrence has been identified as an HRQoL-reducing factor, independently of disease histology (48), and progressive disease is also associated with reduced functional status and HRQoL (55). Recent US data for malignant glioma (including anaplastic astrocytoma, anaplastic oligodendroglioma, glioblastoma, and gliosarcoma) showed that patients with radiographic disease progression performed worse regarding remembering, fatigue, and weakness as well as walking, work, activity, and self-care than patients with stable disease (70). A deterioration was also seen in patients with progression regarding mobility, self-care, and activities. Fatigue was the symptom most often worsened with disease progression, while general activity was the most frequently worsened function (70). Beyond the direct effect of limiting, low functional status may be associated with low levels of resilience, which can make it more challenging for patients to recover and adapt to changes in circumstances linked to their disease (71).

###### 4.2.1.1.2 HRQoL trajectory

Over time, HRQoL may be stable or even improve in low-grade glioma. Dutch data covering the experience of patients with diffuse low-grade glioma over more than 20 years showed that ≥86% of patients maintained or improved their physical and mental HRQoL between 7–26 years after diagnosis, while neurocognitive functioning remained stable or improved in 83% of patients (49). Still, depression and fatigue persisted in 23% and 53% of patients, respectively. Further evidence for stable HRQoL in low-grade glioma was provided by Drewes et al. (56), albeit for a much shorter time horizon. At 6 months after surgery, HRQoL as measured using the EQ-5D 3L was stable in patients with low-grade glioma. In comparison, there was a statistically significant reduction in HRQoL in patients with high-grade glioma, although the authors noted that grade-labelled groups masked different patient HRQoL trajectories. Mood disorders were also more frequent in high-grade relative to low-grade glioma, albeit due to different determinants: chemotherapy and radiotherapy were associated with mood disorders in low-grade glioma and with higher age in high-grade glioma (but not *vice versa*), with cognitive deficits associated with mood disorders reported in both groups (57).

However, these comparative results should not be understood to imply a low humanistic burden in low-grade glioma in absolute terms – impaired physical, cognitive, and emotional functioning are observed in 35%, 50%, and 48% of patients, respectively, within 5 years of treatment (72). Physical, cognitive, and emotional functioning and QoL in patients with low-grade glioma remains below age-matched general population levels (73).

In glioblastoma, for which follow-up data are naturally shorter given patients’ reduced survival, the evidence for HRQoL and functional status are mixed. In patients based in Sweden, the mental component score of the SF-36 at 6 months surpassed the pre-surgery and 3-weeks post-surgery level, while the physical component score was lower at 6 months than pre-surgery (53). Anxiety and depression were more often probably or possibly present at 6 months compared with before surgery.

##### 4.2.1.2 Caregivers

Glioma imposes a humanistic burden not only on patients but also on their caregivers, ranging from feeling helpless and burnt out to negative effects of the disease on the relationship with the patient (74, 75).

In a cohort study from Sweden, Ståhl et al. (76) documented that relatives of patients with glioblastoma scored worse than patients on measures of mental HRQoL and anxiety and depression symptoms. Relatives were more likely to suffer from poor mental HRQoL if their physical HRQoL was impaired or if patients had poor functional status or high levels of anxiety. In a subsequent publication, relatives were shown to suffer from worse mental and physical health over most of the patient’s glioma trajectory and, like patients, to experience worsening of HRQoL over time (53). These findings led the authors to advocate for screening the HRQoL of relatives of patients with glioblastoma from before surgery and offering them support, thereby indirectly also supporting patients who rely on their caregivers.

Such psychological support, however, is not always offered, even if patients report psychological symptoms such as loneliness, rage, depression, or fear of death, as demonstrated in a survey of patients with glioma and their caregivers in Italy (77). In the survey, patients expressed concerns about their role in the decision-making process, recognizing the importance of expressing their wishes regarding treatment options while having insufficient knowledge about medical treatments. Caregivers, in turn, frequently increased their burden by making medical decisions on behalf of patients, even when not confident in their decision, thereby increasing their anxiety levels. Both patients and carers identified symptoms management and rehabilitations as key to returning to normal life and assigned equal importance to physical symptoms such as paralysis or bowel and bladder incontinence and cognitive symptoms such as personality changes and memory loss (77).

#### 4.2.2 Burden of treatment

The currently available literature on the HRQoL effects of specific glioma treatments suggests no significant differences in HRQoL between treatments. In a longitudinal study of multimodal treatment for glioma, no treatment was associated with significant deterioration of long-term cognitive functioning or HRQoL (except a decline in selective attention among patients treated with chemotherapy alone) (78).

Treatment-specific studies also failed to identify differences between most pairs of treatment, including for lomustine-TMZ versus TMZ monotherapy in patients with *MGMT*-methylated glioblastoma (59) or lomustine versus the tyrosine kinase inhibitor regorafenib in patients with recurrent glioblastoma (58). In low-grade glioma, no significant difference in HRQoL was observed between radiotherapy and TMZ (60).

## 5 Treating glioma

### 5.1 An overview of key treatment options for glioma

#### 5.1.1 Surgery

Surgical resection of the tumor is typically the first step in treating all types of glioma and may be repeated, if feasible, for progressed disease (79). A more complete extent of tumor resection as well as lower residual tumor volumes are associated with reduced progression and seizure risk and longer OS in low-grade glioma and glioblastoma, although the level of evidence is low to moderate only (80–85). The idea underpinning resection has been that of “maximum safe resection” – removing the tumor to the greatest extent possible while avoiding new neurological deficits, with neurological deficit prevention prioritized over resection extent (86, 87).

Maximum safe resection has been interpreted differently by different surgeons, leading to definitions of resection extent that have been handled inconsistently in clinical practice (88). A key operationalization of maximum safe resection is gross total resection, commonly understood as the complete removal of the contrast-enhancing tumor mass. Different studies, however, have used the term to refer to reductions of the contrast-enhancing (CE) tumor from 90% to 100% in supratentorial glioblastoma, a range likely including clinically meaningful differences (82). Only in 2021 were evidence-based recommendations on consistent definitions of resection extent proposed (the *Response Assessment in Neuro-Oncology [RANO] resect* classification), which defined, for example, “complete resection” as a resection extent of 100% for contrast-enhancing (in supratentorial glioblastoma) and T2-weighed fluid-attenuated inversion recovery-hyperintense (in supratentorial gliomas of WHO grade 2 and 3) tumors (82). The recommended categories are highly prognostic for clinical outcomes, including for secondary resection in recurrent glioblastoma, and suggested for use in clinical trials (89, 90).

Resection beyond the CE tumor, termed “supratotal” or “supramaximal” resection, has become an active field of research in the last decade, based on the premise that removing non-contrast enhancing tumor or even tissue appearing normal on magnetic resonance imaging (MRI) would capture residual tumors (82, 88). The currently available evidence on the benefits and outcomes associated with supramaximal resection remains mixed. Validation exercises for the RANO *resect* classification showed that patients with IDH-wildtype glioblastoma classified to have undergone supramaximal resection had superior survival outcomes (90) but, in recurrent and previously resected glioblastoma, supramaximal resection was not associated with longer survival but with more frequent deficits following surgery (90). Favorable outcomes, including recurrence and survival, of supramaximal resection were also reported for low-grade gliomas (91). More broadly, reviews for low-grade gliomas and for glioblastoma suggested that supramaximal resection was effective and safe (88, 92). Further and larger clinical studies are needed for more robust evidence.

#### 5.1.2 Radiotherapy

In the treatment of glioma, external beam radiotherapy is used to achieve tumor control and improve survival with limited neurotoxicity (86, 93). Radiotherapy is usually administered within 3–5 weeks of surgery, at 45–60 Gy and 1.8–2.0 Gy per daily fraction; hypofractionated schedules can be considered in elderly patients and in patients with a poor prognosis, with no difference in survival by fractionation schedule (86, 94–99). Care should be taken not to initiate chemoradiotherapy too early after resection (99). Re-irradiation may be considered for glioblastoma recurrence, but survival remains poor in re-irradiated patients with confirmed IDH wildtype glioblastoma and no survival advantage has been demonstrated (100).

Target delineation principles for radiotherapy in glioblastoma were only recently elucidated in published guidelines (97). The gross tumor volume (GTV) should be delineated, in resected tumors, from the resection cavity and encompass residual enhancing tumor on CE T1-weighted MRI but not peri-tumoral edema. Tumor infiltration can be determined from T2 FLAIR, although it was acknowledged that this might be challenging to distinguish from edema. The clinical target volume (CTV) was recommended to be calculated from the GTV and a 15 mm margin for microscopic disease. The planning target volume should then also account for uncertainty in radiotherapy delivery, with a preference expressed for intensity-modulated radiotherapy and volumetric modulated arc therapy due to their improved conformity (97).

Clinical guidelines generally recommend radiotherapy with concurrent or sequential chemotherapy, including for grade 2 and grade 3 IDH-mutant, 1p/19q co-deleted oligodendroglioma and IDH-mutant astrocytoma (**Table 2**), given the benefits of chemoradiotherapy over mono-radiotherapy (79, 94, 95). The long-term neurocognitive side effects associated with radiotherapy and chemoradiotherapy for glioma remain uncertain, although there is some evidence for an increased risk of neurocognitive damage associated with radiotherapy (109).

**Table 2:** Summary of treatment guideline recommendations for glioma.

Grade	First-line therapy	Second-line therapy	Role of lomustine
*Astrocytoma, IDH-mutant*			
2	Radiotherapy + PCV or TMZ (79, 86, 87, 101–103)	• Nitrosoureas • TMZ • Re-resection (86, 87, 101, 103)	Part of first-line treatment (PCV) and as second-line monotherapy
3	Radiotherapy + TMZ (79, 86, 87, 101–105)	• Lomustine or PCV (101) or nitrosoureas in general (86) • TMZ rechallenge	In second-line therapy, as monotherapy or part of PCV
*Oligodendroglioma, IDH-mutant and 1p/19q-codeleted*			
2	Radiotherapy + PCV (TMZ in case of toxicity concerns) (86, 102, 103, 106)	• Chemotherapy, possibly with radiotherapy (depending on prior radiotherapy), mainly TMZ • BSC (102, 106)	Part of first-line treatment (PCV)
3	• Radiotherapy + PCV or TMZ • Radiotherapy (without chemotherapy) • TMZ (86, 102, 103, 106)	• Chemotherapy, specifically lomustine (including as rechallenge (79, 101)) or TMZ • Radiotherapy • Surgery • Systemic therapy (86, 87, 101, 106)	Part of first-line treatment (PCV) and as second-line monotherapy (including as rechallenge)
*Glioblastoma*			
4	• Radiotherapy + TMZ ± TTF (86, 87, 102–104, 107) • Radiotherapy alone may be considered in patients with *MGMT*-unmethylated disease or the elderly/unfit • Radiotherapy + lomustine + TMZ ± TTF may be considered in fit younger patients with *MGMT*-methylated disease (96, 108)	• Nitrosoureas, mainly lomustine • (Repeat) radiotherapy • TMZ rechallenge • Bevacizumab (86, 87, 96, 106)	Potentially part of first-line treatment in fit, younger patients with *MGMT*-methylated disease and standard of care second-line treatment

BSC, Best Supportive Care; IDH, Isocitrate Dehydrogenase; MGMT, O-6-Methylguanine-DNA Methyltransferase; PCV, Procarbazine, Lomustine, Vincristine (combination regimen); TMZ, Temozolomide; TTF, Tumor Treating Fields; WHO, World Health Organization.

#### 5.1.3 Pharmacotherapy

Before reviewing pharmacotherapeutic recommendations, it is worth noting that methylation of the *MGMT* promoter is the key prognostic factor for treatment success, in particular (but not only) for chemotherapy, and for survival (3, 86, 110). When the *MGMT* gene is silenced by promoter methylation, MGMT expression is lost. The subsequent reduction in DNA-repair activity has been linked to increased benefits from therapy with alkylating agents (111). The prognostic value is especially valuable in elderly and/or frail patients, in whom the *MGMT* promoter methylation status should always be assessed before deciding on therapy, as patients without methylated tumors may benefit to a much smaller extent from tumor-specific treatment and chemotherapy (112). For example, in a cohort of patients aged ≥80 years with IDH-wildtype glioblastoma, tumor-specific therapy conferred a median OS benefit of 2.1 months versus best supportive care (BSC), but only in patients with *MGMT* promoter methylation (median benefit of 3.6 months) while no benefit was observed in patients without *MGMT* promoter methylation (113). These and similar data strongly suggested that therapy be initiated dependent on *MGMT* promoter methylation status, which is already reflected in some treatment guidelines (**Table 2**).

##### 5.1.3.1 Chemotherapy

Pharmacotherapy for glioma is currently predominantly chemotherapy using alkylating-based agents. The most used agent is TMZ, due to its good safety profile, although hepatic function needs to be monitored in patients receiving TMZ (86). Nitrosoureas such as lomustine or nimustine have a comparatively less favorable safety profile and may cause cumulative leukopenia and thrombocytopenia, which in turn may lead to treatment interruptions. Lomustine is a particularly interesting case in this treatment class – it serves as the control arm in many clinical trials and is the *de facto* standard of care for recurrent glioblastoma (114).

Lomustine is the second most widely used chemotherapeutic agent, after TMZ, and is administered, with adequate antiemetic prophylaxis, either as a monotherapy or in combination with procarbazine and vincristine (with the combination referred to as PCV) (114). PCV is often preferred over TMZ for IDH-mutant oligodendrogliomas and CNS WHO grade 2 IDH-mutant astrocytoma and *vice versa* for CNS WHO grade 3 IDH-mutant astrocytoma (79).

PCV was first investigated in 1975, for malignant brain tumors, based on the premise that a combined regimen might outperform its constituent treatments given as monotherapy (115). In this phase 2 study, patients were treated in 28-day cycle, which started with oral CCNU (75 mg/m^2^ body surface) on the first day. On days 1 and 8, vincristine was administered intravenously at a dose of 1.4 mg/m2 body surface, while procarbazine (100 mg/m^2^) was administered orally on days 1–14. This study found no benefit of PCV relative to procarbazine alone and to the nitrosourea derivate BCNU in patients treated with partial resection or radiotherapy for primary or metastatic brain tumors and in patients with radiographically and clinically confirmed thalamic masses, metastatic tumors, or brainstem gliomas. Subsequent studies, however, yielded more positive results, including prolonged survival and time to progression with PCV relative to BCNU after radiotherapy for anaplastic gliomas (116) as well as long progression-free survival (PFS) and low rates of histological progression when used as a monotherapy for WHO grade 2 oligodendroglioma (117).

Currently used doses and schedules tend to differ between institutions for the procarbazine and lomustine components while vincristine is generally dosed, on the first day of a cycle, at 1.4 mg/m2 although it may be capped, e.g., at 2 mg, if used in multiple cycles (118). Regarding procarbazine, 150 mg on days 2–11 of a 42-day cycle are used in an UK institution for adjuvant treatment of grade 2–3 gliomas and as palliative treatment for recurrent/progressive low-grade or high-grade gliomas previously treated with TMZ (119), while guidance for a British Columbian institution suggested 60 mg/m^2^ per day for days 2–15 (118). For lomustine, the latter guidance suggested 110 mg/m^2^ on the first day of a cycle, while the former guidance specified 160 mg, also on the first day of a cycle (118, 119). These two examples illustrate the use of similar but still different dosing schedules for PCV components between institutions.

##### 5.1.3.2 Immunotherapy and targeted therapy

Unlike for other cancers, immunotherapy and targeted therapy currently play but a minor role in treating glioma. The VEGF/VEGR inhibitor bevacizumab is the only anti-angiogenic agent approved, for recurrent glioblastoma (in the US but not in the European Union [EU]) and may confer some benefits in PFS and cognitive performance but does not improve OS (120, 121).

Among targeted therapies, vorasidenib, an *IDH1/2* mutation inhibitor, was recently shown to improve PFS and delay the time to next intervention, relative to placebo in patients with IDH-mutant grade 2 glioma, which may suggest considerable potential to widen the therapeutic options for IDH-mutant low-grade glioma (122). Regorafenib was initially also hoped to improve survival in recurrent glioblastoma versus lomustine, based on promising results of the REGOMA trial (123). This benefit, however, was not subsequently confirmed in the GBM AGILE platform trial of regorafenib, which was stopped for futility (124). Larotrectinib is also gaining some interest as a potential targeted therapy although large-scale studies specific to glioma are still missing (125).

For immunotherapies and targeted therapies, several clinical trials can be expected to report in the coming years on targeted therapies for a range of glioma types. However, meaningful improvements, in particular for OS and glioblastoma, should not necessarily be expected if history is any guide – of eleven trials conducted between 2005–2022 for glioblastoma, for example, only three reported a statistically significant OS benefit associated with the investigated treatment (126).

Even in the absence of solid evidence documenting their benefits, several immunotherapy and targeted therapy options are used off-label. Despite not improving OS, bevacizumab is used, off-label, in the EU, with 0.1%, 6.0%, and 59% of patients in the neo-adjuvant, first-line, and second-line setting, respectively, receiving bevacizumab, with its use particularly prevalent in second-line treatment in Spain and France (127). In the US, as many as 5.9% of patients with glioblastoma receive off-label treatment with targeted therapies, with *BRAF*V600E as the most frequent molecular alteration to provide a rationale for targeted therapy use (128). Given the high costs of many targeted therapies and the lack of evidence supporting their widespread use, their case-by-case use is supported by special reimbursements structures. In Italy, for example, the Agenzia Italiana del Farmaco [AIFA] National Fund (“5% Fund”) can be applied to, on a per-patient basis, to cover the costs of BRAF/MEK inhibitors for *BRAF*V600E-mutated glioma (129–131). Similarly, also in Italy, AIFA Managed Entry registries could be employed to monitor the use of drugs such as larotrectinib regarding the link between outcomes and costs (132). Care should, however, be taken to avoid that substantial costs are incurred for relatively few patients and drugs not otherwise meeting efficacy and effectiveness standards to prevent an imbalance in allocating the (finite) resources of a healthcare system.

### 5.2 Treatment recommendations

A wide range of institutions and societies publish treatment guidelines on glioma or specific subtypes, including in Europe (86, 87, 101, 104), the US (79, 102, 103), China (106), and South Korea (105, 107, 133, 134) as well as joint guidelines (96, 135). In addition, guidelines and recommendation papers are available for specific therapies such as radiotherapy (94) or resection (82). With surgery as the first step in glioma treatment, radiotherapy and pharmacotherapy form a large part of guideline recommendations on first- and second-line therapy for astrocytoma, oligodendroglioma, and glioblastoma.

#### 5.2.1 Astrocytoma, IDH-mutant

For grade 2 astrocytoma, incomplete first-line resection and patient age are key drivers of treatment decisions (79, 86, 102). Radiotherapy with PCV is the standard of care based on results of the RTOG 9802 trial that demonstrated PFS and OS benefits of combined therapy versus radiotherapy alone in low-grade glioma (102, 106, 133, 136). TMZ can be offered as an alternative. If a grade 2 astrocytoma progresses, therapy options include re-resection, radiotherapy (if not previously performed), or chemotherapy based on alkylating agents, including nitrosoureas or TMZ (86, 106). Regarding lomustine, these recommendations imply a role as part of first-line treatment with PCV and as second-line monotherapy.

For grade 3 astrocytoma, radiotherapy followed by PCV or maintenance TMZ are the standard of care, based on the CATNON trial that demonstrated an OS benefit with adjuvant TMZ (86, 102, 104, 105, 114, 137). As for grade 2 disease, therapy for recurrent disease depends on prior therapy and patient performance status, with options including lomustine, PCV, and nitrosoureas in general, bevacizumab, or TMZ rechallenge (86, 101). Regarding lomustine specifically, these recommendations imply a role mainly as second-line treatment, either as part of PCV or as a monotherapy.

#### 5.2.2 Oligodendroglioma, IDH-mutant and 1p/19q-codeleted

First-line treatment recommendations for grade 2 oligodendroglioma are as for grade 2 astrocytoma, i.e., specify radiotherapy with PCV (or TMZ in case of toxicity concerns), based on the same set of decision drivers (79, 86, 102, 103, 106). This recommendation is again based on the survival benefits demonstrated for combined therapy in the RTOG 9802 trial (136). Second-line therapy is similar to that for grade 2 astrocytoma, focused on chemotherapy – mainly TMZ and possibly combined with radiotherapy – or BSC (79, 86, 102, 106, 133). The recommended role of lomustine for grade 2 oligodendroglioma is therefore as part of first-line treatment with PCV.

For grade 3 oligodendroglioma, first-line therapy is radiotherapy with either PCV or TMZ, alternatively, although limited to special cases, radiotherapy alone or TMZ alone (86, 102–104, 106). Second-line therapy is recommended to be lomustine (including as a rechallenge) or TMZ (79, 86, 101). Alternatives include radiotherapy, surgery, and systemic therapy, as well as enrollment in clinical trials (86, 87, 101, 105, 106). Based on these recommendations, lomustine is to be considered both as part of first-line treatment (PCV) and as a second-line monotherapy, including as a rechallenge.

#### 5.2.3 Glioblastoma, IDH-wildtype

The recommended first-line treatment for glioblastoma is radiotherapy with concurrent, then maintenance TMZ, with the option to use hypofractionated radiotherapy in unfit and elderly patients (79, 102, 104, 106). These recommendations are based on a 2005 clinical trial by Stupp et al. (138), which showed a median gain of 2.5 months for chemoradiotherapy over radiotherapy alone, with minimal incremental toxicity. Notably, chemoradiotherapy may be effective in elderly patients only for *MGMT*-methylated tumors, while unmethylated tumors in these patients should be treated with radiotherapy only as adding chemotherapy such as TMZ was not found to be more effective (86, 139). In addition to radiotherapy and TMZ, patients with newly diagnosed glioblastoma may be treated with tumor treating fields (TTF), which rely on low-intensity electric fields to generate antimitotic effects on the dividing tumor cell as well as immune modulatory effects (102, 104, 106, 140).

For the treatment of recurrent glioblastoma, lomustine has emerged as the *de facto* standard of care (114), but most guidelines refrain from clear recommendations for the treatment of recurrent glioblastoma, due to a lack of evidence on the absolute and relative merits of different treatment options (86, 102, 104). Listed alternatives to lomustine include other systemic chemotherapy, bevacizumab, re-irradiation, the participation in clinical trials, or BSC (101, 102, 106, 107).

Lomustine, based on these recommendations and considerations, plays a key role in the pharmacological treatment of glioblastoma. It may be considered as a first-line therapy, combined with radiotherapy, TMZ, and possibly TTF in fit, younger patients with *MGMT*-methylated tumors (96, 108). It is the standard of care for recurrent glioblastoma.

### 5.3 Treatment patterns

Real-world treatment patterns showed suboptimal care for glioma in many lower-middle, upper-middle, and high-income countries across histologies (**Table 3**).

**Table 3:** Real-world glioma treatment patterns.

Study context	Treatment patterns
China (141); diagnosed 2006–2019; glioblastoma; n=1,010	• 31% with GTR; 54% with subtotal resection; 15% with biopsy • Only 51% of patients received radiotherapy with adjuvant TMZ, although the proportion increased over time
Denmark (28); data from 2009 to 2014; glioblastoma; n=1,364	• Full post-surgical regimen (radiotherapy with concomitant and adjuvant TMZ) initiated in 50% of patients • 22% of patients without post-surgical patients
Germany (142); diagnosed 1999–2014; glioblastoma; n=40,138	• Surgery (82%) and radiotherapy (75%) most frequent treatments • Chemotherapy use increased over time, from 28% in 1999–2005 to 60% in 2011–2014 (49% overall) • 5% of cases without cancer-related treatment, 10% with surgery only
France (14); diagnosed in 2008; glioblastoma; n=1,856	• 59% received radiotherapy + TMZ, the remainder received radiotherapy only after surgery • Of those with radiotherapy + TMZ, 45% had adjuvant treatment, of which 49% completed ≥6 cycles of adjuvant TMZ • 20% of patients received no cancer-related treatment post-surgery
France (143); diagnosed 2008–2017; high-grade IDH-mutant gliomas; n=1,433	• 72% of the elderly patients received ≥6 cycles of TMZ, 56% received ≥6 cycles of PCV • 87% of patients had a PCV dose reduction, compared with 19% in those receiving TMZ • Nearly half (49%) of patients were prescribed steroids after surgery
Saudi Arabia (144); treated 2008–2018; glioblastoma; n=59	• 39% received concurrent chemoradiotherapy followed by adjuvant chemotherapy, while 37% received only radiotherapy • Of patients receiving adjuvant chemotherapy, 52% received <6 cycles
South Korea (145); diagnosed 2000–2010; WHO grade II gliomas; n=555	• 36% of patients with GTR, 28% subtotal, 13% partial, 24% biopsy • Reduction in chemotherapy use over time, regardless of resection extent • Increased TMZ use versus PCV • 30-40% of patients with non-GTR resection without follow-up treatment
Switzerland; diagnosed 2005–2009 (n=264) (146), diagnosed 2010–2014 (n=310) (147)	• 18.4% of patients received no therapy after surgery in 2010–2014, a slight improvement from 19.5% in the period 2005–2010; higher proportions received monotherapy with radiotherapy (19.1% versus 16.5%) or chemotherapy (7.3% versus 3.4%) while the proportion with radiotherapy with TMZ decreased (45.8% versus 60.6%) • At first progression, no salvage therapy was administered in 40.8% of patients in 2010–2014 (decrease from 54.4% in 2005–2009)
Ukraine (148); diagnosed 2015–2019; glioblastoma; n=2,973	• 61% of patients received radiotherapy or chemotherapy but only 19% received chemoradiotherapy • 39% received surgery as their only treatment
United States (39); diagnosed 2007–2013; glioblastoma; n=4,308	• 50% received systemic therapy, 28% only radiotherapy, 22% no cancer treatment • Most common first-line treatment was TMZ (83%), most common second-line and third-line treatment was chemotherapy with bevacizumab (second-line: 41%, third-line: 57%)
United States (149); diagnosed 2004–2016; anaplastic astrocytoma; n=5,980	• 74% with chemoradiotherapy post-surgery, 8% received only radiotherapy; 15% without adjunct therapy • Chemoradiotherapy use increased over time; no other treatment became less used

PCV, Procarbazine, Lomustine, Vincristine (combination regimen); TMZ, Temozolomide.

#### 5.3.1 Glioblastoma

Most data were available for patients with glioblastoma who were often not treated intensely enough. Firstly, surgery and gross total resection rates were surprisingly low in some countries. In Germany, only 82% of patients with glioblastoma were treated with surgery (142) while in China, fewer than one in three patients achieved gross total resection while 15% of patients had a biopsy only (141). Notably, 5% and 22% of patients with glioblastoma in Germany and the US, respectively, did not receive any oncologic treatment (39).

Secondly, and more commonly, usage rates for radiotherapy with concurrent and adjuvant chemotherapy after surgery were low. Examples included Germany (142), where 10% of patients received only surgery and despite improvements still only 60% of patients received chemotherapy, Denmark (28), where the guideline-recommended protocol combining radiotherapy with concomitant and adjuvant TMZ was initiated in only 50% of patients, and China (141), where only 51% of patients received radiotherapy with adjuvant TMZ. In Switzerland, 18.4% of patients with glioblastoma received no further treatment after surgery in the period 2010–2014, which represented a slight improvement over the period 2005–2009 (19.5%), while salvage therapy was not administered upon progression in 40.8% (2005–2009: 54.5%) of patients (146, 147) (**Table 3**).

Thirdly, if chemotherapy was used, completion of ≥6 cycles was often not achieved. In France, 51% of patients with adjuvant treatment did not complete six cycles of TMZ treatment (14), similar to Saudi Arabia, where 52% received fewer than six cycles of chemotherapy (144). Despite some improvements, such as the increasing use of chemotherapy in China and Germany, many patients with glioblastoma are not offered (timely) treatment or not treated in line with recommendations and best practices, despite the known detrimental benefits of suboptimal treatment, such as reduced OS when failing to complete a sufficient number of chemotherapy cycles (14, 142, 144, 146, 147, 150).

#### 5.3.2 Histologies other than glioblastoma

Similar concerns relating to treatment patterns for histologies apart from glioblastoma are apparent, although fewer data were available (**Table 3**). Patients with low-grade gliomas frequently do not receive adequate therapy after surgery, with 30–40% of South Korean patients with grade 2 gliomas without any follow-up treatment after a non-gross total resection (145) and with 15% of US patients with anaplastic astrocytoma not receiving adjuvant therapy (149). As for glioblastoma, chemotherapy was frequently not sustained for ≥6 cycles, which were achieved, for example, by only 72% and 56% of elderly patients in France receiving TMZ and PCV, respectively, for high-grade IDH-mutant gliomas, while 19% and 87% in needed to reduce their chemotherapy dose (143). Given these data, substantial room for improvement remains in the treatment for non-glioblastoma histologies.

### 5.4 Lomustine: efficacy, effectiveness, safety

As the standard of care for recurrent glioblastoma, lomustine was chosen for a closer, illustrative evidence review as part of this article. Lomustine was approved by the FDA, in 1976. The agent alkylates both DNA and RNA and possibly also inhibits enzymatic functions via carbamoylation of amino acids (114, 151). Lomustine is given orally, as a capsule, every 6–8 weeks (114). Lomustine has become a mainstay of glioma treatment without a clinical trial directly comparing it with placebo but based on the evidence generated in multiple clinical studies, in which lomustine frequently serves as the comparator arm. For recurrent glioblastoma, no other treatment has so far been found to be superior to lomustine regarding OS. In the present section, the efficacy, effectiveness, and safety of lomustine and the PCV regimen are reviewed, as a complement to existing reviews (114, 121).

#### 5.4.1 Efficacy and effectiveness

##### 5.4.1.1 Glioblastoma

###### 5.4.1.1.1 Newly diagnosed glioblastoma

In newly diagnosed glioblastoma, lomustine can be considered in first-line treatment for fit, younger patients (**Table 2**) (96). The case for using lomustine in newly diagnosed patients, in combination with TMZ plus radiotherapy, was made by the CeTeG/NOA-09 RCT, which showed improved survival of combining lomustine with TMZ relative to administering only TMZ in patients with *MGMT* promoter methylation (108). In 129 patients from Germany, the median OS was 31.4 months (95% CI 27.7 to 47.1) in patients who received only TMZ, compared with 48.1 months (95% CI 32.6 months to *not assessable*) in patients receiving both TMZ and lomustine (HR 0.60 [95% CI 0.35 to 1.03], p-value=0.0492). The authors urged caution regarding the robustness of results, however, due to the small sample size. Comparing lomustine and TMZ monotherapies directly, the authors of a small cohort study concluded that patients treated with TMZ survived longer than patients treated with PCV (152). Again, the small sample size warrants caution as only nineteen and twenty-six patients received TMZ and PCV, respectively.

###### 5.4.1.1.2 Recurrent glioblastoma

For recurrent glioblastoma, RCTs including lomustine as a control arm were reviewed and summarized by Weller and Le Rhun (114), who reported low objective response rates (ORRs) – estimated by a recent review at 7.59% when pooled across past clinical trials (153) – and PFS <2 months with lomustine based on these trials. Survival benefits were concentrated in patients with *MGMT* promoter methylation. Importantly, the observed 6-month PFS was approximately 20%, which currently serves as a benchmark for trial planning, and none of the investigational drugs were superior to lomustine. A subsequent network meta-analysis (NMA) of thirty-four RCTs and eight non-randomized studies confirmed the absence of a treatment superior to lomustine (121). Reliable evidence was available for the first recurrence, for which no OS difference was observed for lomustine versus fotemustine, galunisertib, bevacizumab plus lomustine, bevacizumab monotherapy, and bevacizumab plus irinotecan (154). There was low-certainty evidence available for an OS benefit of combined TMZ and ABT414 over lomustine at the time the NMA was conducted, but this benefit was not subsequently confirmed in the full trial publication (155). Similarly, the survival benefit of regorafenib over lomustine was not ultimately confirmed (123, 124).

##### 5.4.1.2 High-grade gliomas

For high-grade gliomas, evidence was available on lomustine as part of PCV. A 2017 Cochrane review identified two RCTs that investigated PCV as a treatment for recurrent high-grade glioma, although only one was ultimately considered due to a lack of statistical power in the other (156, 157). This trial compared PCV with TMZ over a median follow-up of 12 months. No statistically significant difference in OS was observed between treatments (hazard ratio [HR] 0.91, 95% CI 0.74 to 1.11).

##### 5.4.1.3 Low-grade gliomas

###### 5.4.1.3.1 Radiotherapy and PCV

For low-grade gliomas, the seminal RTOG 9802 (136) RCT demonstrated PFS and OS benefits with PCV in addition to radiotherapy relative to radiotherapy alone in patients with grade 2 astrocytoma, oligoastrocytoma, and oligodendroglioma who had undergone subtotal resection or biopsy (RTOG 9802). The trial enrolled 251 patients, who were followed up for a median of almost 12 years. There was a statistically significant OS benefit in those receiving chemoradiotherapy with PCV (13.3 versus 7.8 years, HR 0.59, p-value=0.003). Ten-year PFS and OS rates were 51% versus 21% and 60% versus 40%, for radiotherapy with versus without PCV. Data from RTOG 9802 further suggested that PCV is beneficial for IDH*-*mutant high-risk low-grade glioma and improves both PFS (non-codeleted: HR 0.32, p-value=0.003; codeleted: HR 0.13, p-value<0.001) and OS (non-codeleted: HR 0.38, p-value=0.013; codeleted: HR 0.21, p-value=0.029) (158). No PFS or OS benefits were observed from adding PCV to radiotherapy in patients with IDH wildtype tumors.

Before RTOG 9802, evidence from RTOG 9402 had already led to the proposal that combined radiotherapy and PCV was an effective treatment in patients with anaplastic oligodendrogliomas and oligoastrocytoma in case of 1p/19 codeletion, with median survival of 14.7 versus 2.6 years (HR 0.36 [95% CI 0.23 to 0.57]) for PCV with radiotherapy versus radiotherapy in codeleted tumors, compared with 7.3 versus 2.7 years (HR 0.40 [95% CI 0.27 to.60]) in non-codeleted tumors (29). Overall, median OS was more than twice as long in patients with codeleted tumors who received PCV versus those who did not (14.7 versus 7.3 years, HR 0.59 [95% CI 0.37 to 0.95], p-value=0.03), while no survival difference was observed for patients with non-codeleted tumors. A subsequent analysis of data from this trial showed that survival benefits of radiotherapy with PCV versus radiotherapy alone were limited to those with IDH-mutant disease (9.4 versus 5.7 years, HR 0.59 [95% CI 0.40 to 0.86], p-value=0.006) (159). No prolonged survival was observed in patients with IDH-wildtype disease (1.3 versus 1.8 years, HR 1.14 [95% CI 0.63 to 2.04], p-value=0.67).

###### 5.4.1.3.2 First-line PCV

Follow-up data from RTOG 9402 and the EORTC 26951 trial (160) demonstrated good long-term (recurrence-free) survival in a substantial proportion of patients receiving first-line PCV for anaplastic oligodendroglial tumors (161). In EORTC 26951 participants, median survival with PCV was 3.5 years compared with 2.6 years without, while 14-year survival rates were 25.1% and 13.4%, respectively. In RTOG 9402 participants, the corresponding median survival was 4.8 years, with 14-year survival of 29.1% and 16.5%, respectively. Both studies showed a larger benefit of adding PCV in patients with 1p/19 co-deletion, including 14.2 versus 9.3 years in EORTC 26951 participants and 13.2 versus 7.3 years in RTOG 9402 participants, for treatment with versus without PCV, respectively.

###### 5.4.1.3.3 PCV vs. TMZ

Multiple studies compared PCV and TMZ. The NOA-04 phase 3 RCT suggested that PCV alone was superior to TMZ alone in patients with the best prognosis, with a median PFS of 9.4 (95% CI 3.18 to *not reported*) years with PCV versus 4.46 (95% CI 2.01 to 7.8) years with TMZ over a median follow-up of 9.5 (95% CI 8.6 to 10.2) years (162). Observational studies also consistently reported better effectiveness outcomes with PCV. In 142 patients in Germany with 1p/19q-codeleted WHO grade 2 oligodendroglioma, treatment with PCV only was associated with the longest PFS (9.1 years), relative to 3.6 years in those receiving TMZ (and 5.1 and 4.4 years, respectively, in wait-and-see-treated and resection-only-treated patients) (117). At the same time, the 10-year risk of histological progression to grade 3 was lowest in the PCV-treated group (9%), compared with 75% in the TMZ-treated group. When combined with radiotherapy, data from forty-eight Latin American patients with 1p/19q co-deleted anaplastic oligodendroglioma showed statistically significantly improved PFS (7.2 versus 6.1 years) and OS (10.6 versus 9.2 years) in patients receiving PCV relative to TMZ (163). Analogous results were reported in a French study in 311 patients with IDH-mutant anaplastic astrocytoma, in whom the 4-year PFS was 70.8% if treated with radiotherapy and PCV and only 53.5% if treated with radiotherapy and TMZ (HR 0.58[95% CI 0.38 to 0.87], p-value=0.0074) (164). For OS, the results favored PCV (84.3%) over TMZ (76.6%), but the difference was not statistically significant (HR 0.57 [95% CI 0.30 to 1.05], p-value=0.0675). A systematic review confirmed that adjuvant therapy with PCV was associated with improved OS and PFS versus TMZ in low-grade glioma but noted that the benefit was driven by patients with 1p/19q-codeleted tumors and *IDH1* mutations (165). Patients with a less favorable risk profile were considered to possibly derive larger benefits from radiotherapy combined with concomitant and adjuvant TMZ. A phase 3 RCT is ongoing that investigates the relative merits of concurrent and adjuvant TMZ versus PCV (NCT00887146).

#### 5.4.2 Safety, tolerability, toxicity

##### 5.4.2.1 Toxicities and their effect on treatment use

The most important toxicity associated with lomustine is thrombocytopenia, and both hematological and gastrointestinal toxicities, which are dose-dependent, frequently require doses to be reduced or delayed or for treatment to be discontinued (114, 166, 167). While the risk of severe adverse events with lomustine for recurrent glioblastoma is not elevated relative to treatment options such as regorafenib and bevacizumab (121), thrombocytopenia still limits the use of lomustine as a salvage therapy. A secondary analysis of EORTC 26101 data suggested that 57% and 66% of patients with recurrent glioblastoma who received lomustine and lomustine plus bevacizumab, respectively, experienced ≥1 treatment cycle with thrombocytopenia (168). Delays and reductions in dose and treatment discontinuation were most often due to thrombocytopenia – 8.8% and 10.4% of lomustine and bevacizumab plus lomustine cycles were delayed for any reason, of which 5.0% and 4.9%, respectively, that were delayed due to thrombocytopenia. For dose reductions, the corresponding data indicated reductions in 17.7% and 13.4% of cycles, including 6.3% and 7.1% due to thrombocytopenia, for lomustine and lomustine with bevacizumab, respectively. Lomustine was discontinued in 7.1% of lomustine-treated and 13.4% of lomustine with bevacizumab-treated patients. Overall, 16.0% and 32.9% of patients, respectively, had to modify their lomustine use due to thrombocytopenia, with 26.2% and 49.8% of patients overall having to modify their lomustine use. Based on these findings, the authors argued that efforts to reduce thrombocytopenia could improve the exposure to lomustine and thereby clinical outcomes in patients with recurrent glioblastoma and *MGMT* promoter methylation (168).

A much rarer toxicity associated with nitrosoureas is lung toxicity, including pulmonary fibrosis, for which most evidence pertaining to lomustine comes from case reports from the 1980s. While clinical trials do not routinely report lung fibrosis rates, Seliger et al. (169) noted an uncertainty in clinical practice regarding the necessity for lung function monitoring. In their recently published analyses, they reported no overall increases in pulmonary restriction parameters in 166 patients with recurrent and progressive glioma across multiple lomustine cycles (169). Consequently, lung function monitoring was suggested to be limited to lomustine-treated patients at risk of lung disease due to a prior lung disease or due to risk factors such as smoking or symptoms of worsening lung function.

##### 5.4.2.2 PCV-associated toxicities and their effect on treatment use

As regards PCV, multiple studies have reported significant toxicity with PCV treatment and a subsequent detrimental impact on treatment (**Table 4**). A 2014 Cochrane review identified improvements in OS associated with PCV but noted its association with grade 3 and 4 toxicities in an analysis sample of 900 patients (179). More recently, Keogh et al. (174) reported that for patients with low-grade glioma in Ireland who had undergone surgery and radiotherapy, a median of three, two, and four completed cycles for procarbazine, lomustine, and vincristine, respectively – only 10% of patients were able to complete six PCV cycles without dose modification. Unsurprisingly, dose intensity declined over time, from 98% to 46% with procarbazine, 94% to 48% with lomustine, and 93% to 50% with vincristine, between cycles 1 and 6. Grade 3/4 thrombocytopenia was observed in 14% of patients, grade 3/4 neutropenia in 31%. Similar results were reported for oligodendroglial gliomas in French who received radiotherapy with PCV as their first-line treatment (176). In this retrospective study, 13.4% of patients discontinued PCV due to toxicity, while delayed cycles and reduced doses were reported for 62% and 70%, respectively. Grade 3 toxicity associated with PCV occurred in 38% of patients, grade 4 toxicity in 8%.

**Table 4:** Safety and toxicity data for PCV.

Study context	Glioma, n	Safety, toxicity findings
Ahn et al. (170); South Korea; retrospective analysis of EMR	Recurrent glioma; n=44	*Toxicity (grades 3 and 4), %:* • Anemia: 13.6 • Neutropenia: 18.2 • Thrombocytopenia: 31.8 • Elevated liver enzymes: 13.6 • Elevated creatinine: 2.3
Blonski et al. (171); France; retrospective analysis of EMR	Diffuse low-grade glioma, n=20	*Dose intensity:* • Median of four PCV cycles, only 40% of patients completing six cycles • Four patients (20%) discontinued due to hematological toxicity *Toxicity*: • Grade 3/4 hematological toxicity: 45% of patients
Brandes et al. (172); Italy; retrospective analysis of EMR	Anaplastic astrocytoma, n=49	*Dose intensity:* • Median three PCV cycles • Dose reductions: procarbazine withheld in three patients (skin reaction), vincristine reduced to 50% in 31.9% of patients due to neurological toxicity, procarbazine and lomustine reduced by 25% in 18% of cycles • Chemotherapy interrupted due to toxicity in 37% of patients *Toxicity*: • Grade 3/4 hematological toxicity: 9% of cycles
Carvalho et al. (173); Portugal; retrospective analysis of EMR	Recurrent glioblastoma, n=19	*Dose intensity*. • Chemotherapy discontinued in four patients (21%) *Toxicity, %*: • Grade 3/4 52.6 • Leukopenia: 57.9 • Neutropenia: 47.4 • Thrombocytopenia: 73.7
Esteyrie et al. (164); France; retrospective analysis of POLA database	IDH-mutant anaplastic astrocytoma, n=139	*Dose intensity*: • Dose decreased due to toxicity: 87.1% • Treatment discontinued due to toxicity: 75.8% *Toxicity, %:* • Any grade 3/4 toxicity: 46.7 • Grade 3/4 hepatotoxicity: 19.6 • Grade 3/4 hematotoxicity: 26.7
González-Aguilar et al. (163); Mexico; retrospective analysis of EMR	Anaplastic oligodendroglioma, 1p/19q-codeleted, n=21	Grade 3/4 toxicity: 42.8%
Jutras et al. (166); Canada; retrospective analysis of EMR	Low-grade glioma, n=57	*Dose intensity* • Delays attributable to PCV toxicity: 45.6% of patients • Dose reduction: 21.1% of patients • ≥1 incomplete cycle or treatment discontinued: 38.6% of patients *Toxicity, %*: • Grade 3 anemia: 7.0 • Grade 3 neutropenia: 10.5 • Grade 3 thrombocytopenia: 28.1 • Increased aminotransferase: 64.9 • Low CD4 count: 31.6 • Neurotoxicity: 59.6 • Nausea: 70.2 • Vomiting: 40.3
Keogh et al. (174); Ireland; retrospective analysis of EMR	Low-grade glioma, n=41	*Dose intensity* • Six cycles completed: 48% of patients (only 10% without any dose modifications) • Median number of cycles: procarbazine = 3, lomustine = 2, vincristine = 4 • ≥1 dose omitted: cycle 1 = 17% of patients, cycle 6 = 34% • Treatment discontinued by cycles 4 and 6: 15%, 27% (overall, 51% of patients with treatment discontinued prematurely) • Dose intensity from cycle 1 to cycle 6: o Procarbazine: 98% to 46% o Lomustine: 94% to 48% o Vincristine: 93% to 50% *Toxicity*: • Hematological: 66% of patients (grade 3/4 thrombocytopenia: 14%; grade 3/4 neutropenia 31%)
Kristof et al. (175); Germany; retrospective analysis of EMR	Astrocytoma, oligodendroglioma, oligoastrocytomas, n=48	*Dose intensity* • Share with >4 PCV cycles: 37.5% (median number of PCV cycles = 4) • Treatment discontinuation due to toxicity: 21% of patients *Toxicity, %*: • Grade 3/4 leukopenia: 32.5 • Grade 3/4t thrombopenia: 36.5 • Grade 3/4 anemia: 5.5 • Grade 3/4 hepatotoxicity: 15.5 • Infections: 19
Tabouret et al. (176); France; retrospective analysis of EMR	Oligodendroglial tumors, n=89	*Dose intensity* • Completed six cycles: 37% of all patients (13.5% of all patients discontinued due to toxicity, 3.4% due to other complications) • Total doses reduced by 70%, lomustine dose by 52% (85% in those who did not progress) *Toxicity, %* • Any grade 3/4: 39 • Grade 3/4 hematotoxicity: 37 • Grade 3/4 hepatotoxicity: 8
Wick et al. (177); Germany; phase 3 trial (NOA-04)	Anaplastic glioma; n=318	*Dose intensity* • Median (range) number of completed cycles: 4 (1 to 5) • Dose reductions: 16% • Dose delays: 18% of cycles delayed due to hematologic toxicity (for a median 14 days) • Discontinuation: 9% of patients due to procarbazine allergy or hematologic toxicity; 7% due to clinically relevant polyneuropathies in patients receiving vincristine *Toxicity (in patients receiving PCV)* • Grade 3/4 hematologic toxicity: 25% • Allergic reaction: 19.1% • Polyneuropathy: 14.7% • Transaminase elevation: 25%
Webre et al. (178); United States; retrospective analysis of EMR	Primary anaplastic oligodendroglioma, n=76	*Dose intensity* • Dose reductions: 35.5% of all patients • Dose delays: 30.3% of all patients *Toxicity, %* • Grade 3/4 thrombocytopenia: 2.6 • Grade 3/4 leukopenia: 1.3 • Neurotoxicity: 14.5

EMR, Electronic Medical Record; PCV, Procarbazine Lomustine Vincristine (regimen); TMZ, Temozolomide.

Studies ordered alphabetically by surname of the first author.

Some non-randomized studies have suggested that PCV toxicity may be due to vincristine, which has poor bioavailability and cannot cross the blood-brain barrier (114, 167). The most recent of these studies was performed in South Korean patients with recurrent glioma, who had received PCV or procarbazine plus lomustine (PC) and whose data were analyzed retrospectively (**Table 4**) (170). In the PC group, OS was statistically significantly longer than in the PCV group (396 versus 232 days, p-value=0.042) but no difference in PFS was observed (284.5 versus 131 days, p-value=0.077). Hematotoxicity was significantly reduced in the PC group, including for anemia (6.7 versus 45.5%, p-value=0.02) and thrombocytopenia (20.0 versus 70.4%, p-value<0.001). Adverse effects on therapy, including delays, dose reductions, or discontinuations, were also less frequent with PC than with PCV (26.7 versus 68.2%, p-value=0.012). In an earlier retrospective analysis of 145 German patients with oligodendroglial tumors, no statistically significant difference between PCV and PC was found for PFS (HR 0.81 [95% CI 0.53 to 1.25], p-value=0.35) (180). Neurotoxicity was less prevalent in the PC group, but no further differences were observed except for white blood cell count.

In contrast to these findings, data from ninety-seven US-based patients who had received PCV or PC for 1p/19q-codeleted anaplastic oligodendroglioma before first progression were analyzed (178). No difference in OS or PFS was observed between groups, nor was there a statistically significant difference in dose reductions or delays. Neurotoxicity occurred only in the PCV group, but even this difference was not found to be statistically significant. The authors concluded that, over a median follow-up of 9.9 years, initial therapy with PC was comparable to PCV regarding both effectiveness and tolerability (178). The effectiveness of PC was noted to be unsatisfactory, with a median OS from PC administration of 9.7 months (95% CI 6.7 to 12.7) and median PFS of 8 weeks, in a South Korean single-arm trial that enrolled eight patients with *MGMT*-methylated recurrent glioblastoma (181).

##### 5.4.2.3 PCV vs. TMZ

Toxicity comparisons between PCV and TMZ have generally favored TMZ. In the NOA-04 RCT, most allergic reactions (13 of 14) and grade 3/4 hematologic toxicities (14 of 17) in patients with grade 3 anaplastic glioma treated with chemotherapy occurred in patients treated with PCV (n=68) relative to TMZ (n=67) (177). All polyneuropathy (n=10) and elevated transaminase (n=14) events occurred in the PCV arm. Dose discontinuations due to procarbazine allergy or hematologic toxicity occurred in 9% of patients, dose reductions in 16% and 6% of PCV- and TMZ-treated patients, respectively. Polyneuropathy was observed in 10% of vincristine-treated patients, resulting in treatment discontinuation in 7% of patients (177). Non-randomized studies also showed less toxicity with TMZ. A systematic review of randomized and non-randomized studies in low-grade glioma noted that the comparatively better effectiveness of PCV was associated with higher rates of toxicity and subsequent treatment changes (165). A retrospective French cohort study in 311 patients with IDH*-*mutant anaplastic astrocytoma estimated that, following radiotherapy, grade 3/4 toxicities were statistically significantly more frequent with PCV than TMZ (46.7 versus 7.8%, p-value<0.0001) (164). Dose reductions were required in 87.1% of patients receiving PCV, versus only in 23.2% of patients with TMZ (p-value for difference<0.0001). Similarly, an extremely high toxicity-related discontinuation rate of 75.8% was reported for PCV, compared with 39.5% for TMZ (p-value for difference<0.001). In a Spanish retrospective study of patients with 1p/19q-codeleted anaplastic oligodendroglioma, grade 3/4 were also reported to be more frequent in the PCV versus the TMZ group (42.8 versus 11.1%, p-value<0.0016) (163). Less than half (42.8%) of patients completed their PCV protocol, compared with 80.2% of patients completing their TMZ protocol.

Taken together, these findings suggest that lomustine and PCV are associated with toxicities that require careful management to avoid an undue burden on patients and treatment disruptions. For lomustine, suggestions have included the use of drugs such as the thrombopoietin receptor agonist romiplostim that regulate platelet production, to counter thrombocytopenia (114, 167). More generally, recent recommendations on managing complications arising from treating primary CNS tumors have stated that systemic pharmacotherapy should not be considered in patients with depleted neutrophil (≤1,500 μL) and platelet (≤100,000 μL) counts (167). Vincristine exposure should be terminated if there is evidence for polyneuropathy.

## 6 Discussion

The present review documented the burden of glioma for patients, caregivers, and healthcare systems. Survival varies by type, treatment, recurrence, and healthcare system, but is often <2 years and <1 year in glioblastoma.

Gliomas occur relatively infrequently, compared with other cancers, and their incidence exhibits some variation between countries and, in the US, counties (182, 183). Differences in incidence between countries are partly due to different capacities available for case ascertainment and reporting, while differences in survival and mortality likely also reflect differences in healthcare system performance and access (183, 184). Recent trends in particular, which have indicated increases, for example, of glioblastoma incidence, have been attributed to improvements in diagnostic methods such as neuroimaging, including within and between countries (184–186).

However, neither geographic differences nor recent trends are simply due to differences or changes in diagnostic methods, although all underlying mechanisms are not yet known (187). Geographic differences plausibly also result from differences in molecular markers that predispose towards the development of glioma and are associated with survival (188–192). Examples include the more frequent occurrence of human leukocyte antigen variants associated with an increased glioma incidence in Caucasians, which may explain their higher glioma incidence (192), the association between European ancestry and increased glioma risk in African Americans and Hispanics in the US (193) and, on a population level, an increased glioma incidence in predominantly Caribbean-origin counties in the US relative to predominantly Mexican/Central American-origin counties (194). Further elucidation of these and similar relationships would likely be crucial to reducing glioma incidence while improving outcomes. Ideally, such research would be conducted in multidisciplinary settings (190) and expand beyond its predominantly US focus.

Explanations for trends towards increasing glioma incidence are more strongly focused on improvements to diagnosis, classification, and cancer registration, but complementary explanations have also been investigated. Most notably, both widespread environmental and lifestyle changes such as traffic-related air pollution but also the increased of ionizing radiation, e.g., for medical purposes, have been suggested as contributors to the increased incidence of glioma (37, 195).

Despite their relatively low incidence, gliomas are associated with substantial costs, including due to costly procedures such as surgery as well as regular interactions with healthcare providers. Glioma survivors face additional costs from reduced productivity or the need to retire while frequently in medical debt, particularly in the US. Societies are confronted with revenue lost to premature deaths. Furthermore, patients typically have reduced HRQoL due to the disease itself and treatment, and the effect of treatment on QoL has been identified as one of the major concerns for patients when making treatment decisions. Caregivers are similarly affected as they are, or consider themselves to be, responsible for treatment decisions and may be affected by difficult interactions with the patient.

Treatment for grade 2 and 3 gliomas is centered on first-line radiotherapy in combination with PCV or TMZ, while second-line treatment includes chemotherapy (rechallenge), (repeat) radiotherapy, and possibly re-resection (**Table 2**). First-line treatment for glioblastoma is radiotherapy combined with TMZ or lomustine, possibly combined with TTF, based on patient fitness and *MGMT* promoter methylation status. Lomustine monotherapy is the standard of care for recurrent glioblastoma.

### 6.1 Unmet treatment needs in clinical practice

Treatment for glioma, particularly glioblastoma, is beset with challenges, and there is considerable room for improvement, as is evident from the frequently still exceptionally poor survival outcomes. Challenges include repeated disappointments in the development of new treatment options, particularly immunotherapies and targeted therapies, none of which have so far improved on the available therapies (79, 126). For a substantial number of potential treatment choices, evidence is not (yet) available or remains inconsistent, including, for example, uncertainty around managing oligodendrogliomas without initial radiotherapy or the potential interchangeability of PCV and TMZ in these patients (196). The most recent WHO classification increases the complexity of choosing treatments based on evidence, as newly defined glioma types and subtypes may not map directly to past trials that used prior classifications (86).

### 6.2 Under-treatment and room for improving toxicity management

In clinical practice, treatments are often employed suboptimally, as patients with different histologic types and across countries have been reported to receive insufficient treatment (**Table 3**). This includes not receiving any treatment, not being treated with (chemo-) radiotherapy after initial resection, and not completing the (full) chemotherapeutic schedule. Dose delays and reductions and treatment discontinuation are a concern with lomustine, PCV, or TMZ, which can cause a range of primarily hematological toxicities that may make it impossible to complete the planned number of treatment cycles and doses. Improvements to the management of toxicities are therefore urgently needed, and possible, e.g., by monitoring closely for and reacting quickly to the development of thrombocytopenia by administering romiplostim (197).

Toxicities could be limited further by tailoring recommendations for specific treatments to those patients most likely to derive the greatest clinical benefit. The benefits of PCV in oligodendroglioma, for example, are accrued mostly by patients with IDH-mutant and 1p/19q-codeleted tumors, and the benefits of PCV over radiotherapy are limited to IDH-mutant disease (136, 159). The benefits of alkylating chemotherapy in elderly patients with newly diagnosed glioblastoma seem to be limited to patients with *MGMT* promotor methylation (113). These and similar findings have motivated the latest revision of the WHO glioma classification, which incorporates molecular changes that are clinicopathologically useful (at the cost of, as mentioned before, limiting “backwards compatibility” of study data) (7, 86, 198). Guidelines also point out the importance of considering IDH mutation, 1p/19q codeletion, and *MGMT* promoter methylation status (79, 86, 103, 104). It seems plausible that increasing the awareness of healthcare providers about the importance of these markers and treatment recommendations could allow for more targeted treatment that also avoids unnecessarily exposing patients to the risk of toxicity. While increasing guideline awareness is challenging, promising suggestions from the recent literature focused on the combination of more traditional dissemination strategies (such as continuing education) with multilevel social media campaigns that involve guideline authors, clinicians, and patients and provide a more informal way of communicating guideline content (199).

### 6.3 Strengthening the diagnostics and evidence for glioma

Beyond avoiding toxicities, molecular diagnostics may increase the precision of diagnosis and treatment choice. While molecular profiling and targeting are still relatively new in clinical practice for glioma, several recent studies have supported the notion that molecularly guided therapy is feasible and provides clinical benefit, including in glioblastoma and recurrent glioma (24, 200, 201). A guideline on rational molecular testing of gliomas, glioneural, and neuronal tumors also argued for molecular testing to target therapy but cautioned that only a handful of targets had sufficient evidence available to justify testing for them in standard care (202).

Health economics more broadly could be strengthened in the field of glioma to provide healthcare decision-makers, healthcare providers, and patients with evidence beyond efficacy, effectiveness, and safety. In a systematic review of health economic analyses in low-grade glioma, Tuohy et al. (203) concluded that the existing studies were of good quality but limited in number, despite the economic impact of glioma. They argued that more research was required to identify the value of low-grade glioma management strategies. A concern identified in their review pertained to a lack of appropriate health state utility values (HSUVs) to capture the QoL impact of glioma and treatments. As such values are now available (204) or may have been overlooked in previous health economic analyses (85), efforts should be made to ensure that the comparative value of glioma treatments be scrutinized in more depth. For lomustine and PCV, respectively, the available literature is particularly sparse and must be considered at least partly outdated. The few available examples included a 2006 cost-effectiveness analysis showing lomustine to dominate TMZ for malignant glioma in British Columbia as lomustine was associated with prolonged survival at lower cost (205) and a 2017 cost-utility analysis for the US that showed PCV in combination with radiotherapy to be very likely cost-effective relative to radiotherapy only for high-risk low-grade glioma (206).

Improvements to evidence generation, including but not limited to HRQoL, have motivated efforts to define a core outcome set (COS) for glioma trials, which until recently had been lacking. In 2022, Millward et al. (207) argued that a COS for neuro-oncology would harmonize outcome measurement and standardized analysis, interpretation, and reporting in clinical studies but that it would be challenging to strike a balance between a COS that was too specific (e.g., for a subtype) or too broad. At the time, a set of patient-reported outcomes had been developed by the Fast TRACK COA group, which represented learned societies and regulatory agencies from the US and Europe (208). The construct set included four core symptoms (pain, difficulty communicating, perceived cognition, occurrence of seizures, in addition to treatment-related symptomatic adverse events) and two core functional constructs (physical functioning, role functioning). These constructs were recommended for data collection in clinical trials. As of 2023, a first COS is available for phase 3 interventional trials of primary glioma, developed as part of the COBra study (209). This COS includes seven domains (survival; adverse events; activities of daily living; HRQoL; seizure activity; cognitive function; physical function), most of which have subdomains such as severity of adverse events or overall tolerability in the adverse events domain. This and similar tools, as well as collaborative efforts to align different COS internationally, have the potential to ensure that trials generate outcomes that are relevant for all facets of treatment assessment, including clinical, economic, and humanistic evaluations.

### 6.4 Development of novel treatment options

The final, perhaps most obvious but also most challenging, step towards improving care for glioma are novel effective treatments. Recent trial history does not suggest cause for an overabundance of optimism as, for example, both bevacizumab and regorafenib ultimately failed to meet expectations despite initially promising data (123, 124). With these examples in mind, the results recently published from a phase 3 trial of vorasidenib in IDH-mutant low-grade glioma (122) and from a phase 1 trial of vorasidenib and the *IDH1* mutation inhibitor ivosidenib, also in *IDH1*-mutant low-grade glioma (210) pointed to potential treatment improvements for low-grade glioma. In addition, multiple studies have shown vaccines to be safe, including for diffuse midline glioma (211), newly diagnosed high-grade glioma (212), and newly diagnosed and recurrent glioblastoma (213), but no efficacy has been demonstrated. While the benefits and harms of most of these treatments require further corroboration in clinical trials and/or practice, there is the hope that at least some of these treatments will prove helpful in ameliorating glioma care and outcomes and help to reduce the considerable clinical and humanistic burden currently faced by people with glioma.

## Author contributions

JP: Conceptualization, Investigation, Writing – original draft, Project administration. MW: Writing – review & editing, Investigation. AM: Writing – review & editing, Investigation. KG-H: Conceptualization, Writing – review & editing, Funding acquisition, Project administration, Investigation. LK-C: Conceptualization, Writing – review & editing, Funding acquisition, Project administration, Investigation. SR: Conceptualization, Investigation, Writing – review & editing. RP: Conceptualization, Writing – review & editing, Investigation.
