# Supplementary material for: High costs, low quality of life, reduced survival, and room for improving treatment: an analysis of burden and unmet needs in glioma
Source: Front Oncol. 2024 Mar 20;14:1368606. doi: 10.3389/fonc.2024.1368606 (PMC10987841; doi:10.3389/fonc.2024.1368606)
Supplement: Supplementary file 1 [file DataSheet_1.pdf]

Supplementary Material

High Costs, Low Quality of Life, Reduced Survival, and Room for Improving Treatment: An Analysis of Burden and Unmet Needs in Glioma

Johannes Pöhlmann*, Michael Weller, Andrea Marcellusi, Kristin Grabe-Heyne, Lucia Krott-Coi, Silvia Rabar, Richard F. Pollock

*** Correspondence:**
Johannes Pöhlmann
poehlmann@covalence-research.com
Covalence Research Ltd, Rivers Lodge, West Common, Harpenden AL5 2JD, United Kingdom

1 Glioma Survival

Table S1 Glioma survival by type/entity in selected primary and registry-based studies

Study; design	Glioma type/entity	Country; n	Treatment	Survival outcomes
Blakstad *et al.* (1); retrospective cohort study	Glioblastoma, recurrent	Norway; n=467	RT, TMZ	• Overall:<br>○ Median OS, months: 12.1<br>○ Median PFS, months: 8.1<br>○ 2-year OS: 21.4%<br>○ 5-year OS: 6.8%<br>• Resection status, median OS, months:<br>○ Gross total resection: 17.2<br>○ Subtotal resection: 11.4<br>○ Biopsy: 6.8<br>• *IDH* mutation status, 1/2/5-year OS, %<br>○ Mutated: 70.3, 54.1, 12.2<br>○ Wildtype: 53.3, 20.1, 5.5<br>○ Unknown: 28.4, 12.4, 7.2<br>• RT and concomitant and maintenance TMZ at primary diagnosis:<br>○ Median OS, months: 16.1<br>○ 5-year OS: 9.3%
Brown *et al.* (2); retrospective cohort study	Glioblastoma	UK; n=490	Biopsy, surgery	Median OS, months:<br>• All: 9.2<br>• Biopsy, months: 8.0<br>• Debulking surgery: 14.9

Study; design	Glioma type/entity	Country; n	Treatment	Survival outcomes
Cairncross *et al.* (3); phase 3 RCT (RTOG 9402)	Oligodendroglioma, anaplastic	US; n=291	PCV+RT versus RT	Median survival, years: PCV+RT: 4.6 vs. RT: 4.7
Cheo *et al.* (4), retrospective cohort study	Glioblastoma	Singapore; n=107	Chemotherapy, RT	Median OS, months:<br>• Overall: 15.1 (2-year OS: 23.5%)<br>• RT >50 Gy vs. no RT >50 Gy: 16.1 vs. 8.7<br>• Maintenance concurrent chemotherapy vs. no maintenance concurrent chemotherapy: 16.4 vs. 9.2
Claus *et al.* (5); registry study	Astrocytoma	US; n=1,458	-	Median OS, years: 5.2
	Glioma, mixed	US; n=946		Median OS, years: 5.6
	Oligodendroglioma	US; n=421		Median OS, years: 7.2
Decavèle *et al.* (6); retrospective cohort study	Glioma, high-grade; treated in intensive care unit	France; n=78	-	1-year OS: 27%
De Witt Hamer *et al.* (7); registry study	Glioblastoma	Netherlands; n=2,409	Surgery	• 30-day mortality: 5.2%<br>• 2-year OS: 13.5%
Efremov *et al.* (8); registry study	Glioblastoma	Germany; n=40,138	-	• Median (95%) OS, months: 10.0 (9.0 to 10.0)<br>• 2-year OS: 19.3%
Fabbro-Peray *et al.* (9); population-based cohort study	Glioblastoma	France; n=2,053	RT, TMZ	Median (95%) OS, months:<br>• Overall: 11.2 (10.7 to 11.9)<br>• No oncologic treatment: 1.8 (1.6 to 2.1)<br>• Treatment apart from RT+TMZ: 5.9 (5.5 to 6.6)<br>• RT+concomitant TMZ: 16.4 (15.2 to 17.4)<br>• RT+maintenance TMZ: 18.9 (18.0 to 19.8) (in those with ≥6 cycles of TMZ: 25.5 [24.0 to 28.3])

Study; design	Glioma type/entity	Country; n	Treatment	Survival outcomes
Fuentes-Raspall *et al.* (10); registry study	Astrocytoma, anaplastic	Spain; n=56	-	Mean (95% CI) OS, %:<br>• 1 year: 50.0 (38.5 to 65.0)<br>• 5 years: 8.5 (3.4 to 21.2)
	Astrocytoma, diffuse	Spain; n=113		Mean (95% CI) OS, %:<br>• 1 year: 60.2 (51.8 to 69.9)<br>• 5 years: 44.3 (35.9 to 54.6)
	Astrocytoma, pilocytic	Spain; n=35		Mean (95% CI) OS, %:<br>• 1 year: 97.1 (91.8 to 100)<br>• 5 years: 94.0 (86.3 to 100)
	Glioblastoma	Spain; n=463		Mean (95% CI) OS, %:<br>• 1 year: 24.0 (20.4 to 28.3)<br>• 5 years: 3.3 (2.0 to 5.6)
	Oligodendroglioma	Spain; n=21		Mean (95% CI) OS, %:<br>• 1 year: 95.2 (86.6 to 100)<br>• 5 years: 69.1 (51.2 to 93.3)
Giradi *et al.* (11); analysis of global brain tumor data (CONCORD-3)	Astrocytoma, diffuse and anaplastic	Global; n=556,237 (overall)	-	• Age-standardized 5-year net survival: 20% (e.g., China, South Korea, Denmark, Italy) to 38% (e.g., Canada, US, Germany, UK)<br>• Trend towards improved (e.g., Australia, Canada, Netherlands, Northern Europe) or stable survival
	Glioblastoma			• Age-standardized 5-year net survival: 4% (Ecuador) to 17% (China)<br>• Broad trend towards improved survival but fluctuations or declines e.g., in Germany, Ireland, Israel<br>• Consistent improvement in patients aged 40–70 years

Study; design	Glioma type/entity	Country; n	Treatment	Survival outcomes
	Oligodendroglioma			• Age-standardized 5-year net survival: 32% (e.g., Denmark, Poland) to 69% (e.g., Finland, Germany, Slovenia)<br>• Broad trend towards improved survival but fluctuations or decline e.g., in Finland, New Zealand, Taiwan
Gramatzki *et al.* (12,13); analysis of cancer registry data	Glioblastoma	Switzerland; n=264 (diagnosed 2005–2009) and n=310 (diagnosed 2010–2014)	Biopsy, resection, chemotherapy, radiotherapy, bevacizumab	Median (95% CI) OS, months:<br>• 2005–2009 cohort: 11.3 (10.1 to 12.3)<br>○ IDH wildtype: 11.3 (9.4 to 13.2)<br>○ IDH mutant: 60.5 (27.3 to 93.7)<br>○ IDH unknown: 10.7 (9.3 to 12.2)<br>○ Only surgery: 1.9<br>○ Surgery followed by radiotherapy: 6.2<br>○ Surgery followed by TMZ: 6.7<br>○ Surgery followed by radiotherapy and TMZ: 17.0<br>• 2010–2014 cohort: 13.1 (11.5 to 14.6)<br>○ IDH wildtype: 13.5 (11.8 to 15.2)<br>○ IDH mutant: 23.7<br>1-year OS, %:<br>• 2005–2009 cohort: 45.4 (IDH wildtype: 46.4, IDH mutant: 75.0)<br>• 2010–2014 cohort: 55.1 (IDH wildtype: 57.2, IDH mutant: 66.7)<br>2-year OS, %:<br>• 2005–2009 cohort: 21.0 (IDH wildtype: 20.6, IDH mutant: 50.0)<br>• 2010–2014 cohort: 20.2 (IDH wildtype: 18.6, IDH mutant: 50.05)<br>3-year OS, %:

Study; design	Glioma type/entity	Country; n	Treatment	Survival outcomes
				• 2005–2009 cohort: 13.0 (IDH wildtype: 12.5, IDH mutant: 62.5)<br>• 2010–2014 cohort: 12.8 (IDH wildtype: 10.6, IDH mutant: 44.4)<br>5-year OS, %:<br>• 2005–2009 cohort: 7.9 (IDH wildtype: 6.4, IDH mutant: 62.5)<br>• 2010–2014 cohort: 6.2 (IDH wildtype. 4.4, IDH mutant: 44.4)
Hansen *et al.* (14); registry study	Glioblastoma	Denmark; n=1,364	Biopsy, partial or total resection	Median OS, months:<br>• Overall: 11.2<br>• Biopsy: 4.7<br>• Resection, partial: 11.5<br>• Resection, total: 15.6
Hertler *et al.* (15); registry study (ETERNITY)	Glioblastoma, IDH wildtype, ≥5-year survival since diagnosis	Australia, Europe, North America; n=189	-	• PFS, median (95% CI), years: 4.6 (3.9 to 5.3)<br>• OS, median (95% CI), years: 9.9 (7.9 to 11.9)
Marra *et al.* (16); retrospective cohort study	Glioma, high-grade	Brazil; n=47	RT	Median (range) survival, months: 16.6 (4.4 to 67.7)
Mathiesen *et al.* (17); registry study	Glioma, low-grade	Sweden; n=2,012	-	Median (95% CI) OS:<br>• 1 year: 82% (78 to 85) in Sweden, 91% (85 to 95) in Stockholm<br>• 2 years: 72% (68 to 76) in Sweden, 82% (74 to 88) in Stockholm
	Glioma, high-grade	Sweden; n=587	-	Median (95% CI) OS:<br>• 1 year: 34% (32 to 36) in Sweden, 51% (46 to 56) in Stockholm

Study; design	Glioma type/entity	Country; n	Treatment	Survival outcomes
				• 2 years: 14% (12 to 16) in Sweden, 25% (21 to 30) in Stockholm
	Glioblastoma	Sweden; n=1,110	-	Median (95% CI) OS:<br>• 1 year: 29% (26 to 32) in Sweden, 42% (36 to 48) in Stockholm<br>• 2 years: 7% (5 to 9) in Sweden; 13% (10 to 18) in Stockholm
Narita *et al.* (18); registry study	Astrocytoma, diffuse	Japan; n=351	-	• PFS, 5-year: 57.0%<br>• OS, 5-year: 75.0%
	Glioblastoma	Japan; n=1,489		• PFS, 5-year: 9.2%<br>• OS, 5-year: 10.1%
	Oligodendroglioma and oligoastrocytoma	Japan; n=211		• PFS, 5-year: 74.6%<br>• OS, 5-year: 90.0%
	Oligodendroglioma, anaplastic	Japan; n=232		• PFS, 5-year: 28.7%<br>• OS, 5-year: 41.1%
Ostrom *et al.* (19); registry study	Astrocytoma, IDH-mutant, grade 2	US; n=8,561	-	1-year OS: 98.0%
	Astrocytoma, IDH-mutant, grade 3			1-year OS: 92.4%
	Astrocytoma, IDH-mutant, grade 4			1-year OS: 76.3%
	Glioblastoma, IDH wildtype, grade 4			1-year OS: 53.7%
	Glioma, diffuse, midline, H3 K27M			1-year OS: 55.9%

**Study; design**	**Glioma type/entity**	**Country; n**	**Treatment**	**Survival outcomes**
	Oligodendroglioma, IDH-mutant and 1p/19q codeleted, grade 2			1-year OS: 97.9%
	Oligodendroglioma, IDH-mutant and 1p/19q codeleted, grade 3			1-year OS: 94.4%
Picart *et al.* (20); registry and retrospective cohort study	Glioblastoma (cerebellar)	France; n=118	Biopsy, resection	Optimal (total) resection vs. partial resection/biopsy:<br>• PFS, months: 6.5 vs. 4.3<br>• OS, months: 16.7 vs. 6.2
Reiner *et al.* (21); registry study	Astrocytoma (patients aged ≥65 years)	US; n=1,291	-	Median OS (95% CI), months: 5.8 (5.3 to 6.4)
	Oligodendroglioma (patients aged ≥65 years)			Median OS (95% CI), months: 22.6 (13.9 to 33.1)
Shieh *et al.* (22); registry study	Glioblastoma	Taiwan; n=3,732	-	Median (95% CI) OS, months: 12.5 (12.0 to 13.0)
Solomons *et al.* (23); case-note review	Glioma, low-grade	UK; n=153	-	5/10-year, %:<br>• PFS: 93.1, 68.7<br>• OS: 100, 95.8
Tabei *et al.* (24); retrospective cohort study	Glioblastoma at first progression	Japan; n=147	-	Median (95% CI) OS after the first progression, months:<br>• Primary glioblastoma, mutant *IDH1*: 13.5 (3.7 to 23.2)<br>• Primary glioblastoma, wildtype *IDH1*: 10.5 (8.6 to 12.4)<br>• Primary glioblastoma, overall: 10.5 (8.7 to 12.3)<br>• Secondary glioblastoma, overall: 10.1 (5.7 to 14.4)

Study; design	Glioma type/entity	Country; n	Treatment	Survival outcomes
Tesileanu *et al.* (25); retrospective study	Astrocytic glioma, diffuse, *IDH1/2* wildtype with molecular features of glioblastoma and WHO grade 4; *IDH1/2* wildtype glioblastoma	Netherlands; n=87	-	Median OS, months:<br>• Astrocytoma: 23.8<br>○ Astrocytoma, *pTERTmt* only: 14.4<br>• Glioblastoma: 19.2
van den Bent *et al.* (26); phase 3 RCT (CATNON)	Anaplastic glioma, 1p/19q non-codeleted	Australia, Europe, North America; n=751	RT, TMZ (concurrent and/or maintenance)	Concurrent versus non-concurrent TMZ:<br>• Median (95% CI) OS, months: 66.9 (45.7 to 82.3) vs. 60.4 (45.7 to 71.5)<br>• 5-year OS (95% CI): 52.7% (46.9 to 58.1) vs. 50.2% (44.4 to 55.7)<br>Maintenance versus non-maintenance TMZ:<br>• Median (95% CI) OS, months: 82.3 (67.2 to 116.6) vs. 46.9 (37.9 to 56.9)<br>• 5-year OS (95% CI): 58.5% (52.8 to 63.8) vs. 44.3% (38.6 to 49.9)
Vargas López *et al.* (27); retrospective cohort study	Glioma, high-grade (grade 3,4)	Spain; n=233	At progression: salvage surgery	Median (range) survival, months:<br>• Overall: 15.4 (0 to 66.3)<br>• After recurrence: 7.8 (0 to 47.4)
Villani *et al.* (28); prospective observational study	Glioma	Italy; n=99	-	Median (range) OS, months:<br>• Overall: 27 (14.9 to 39.1)<br>• Glioblastoma: 19 (17.3 to 20.8)<br>• No glioblastoma: 145 (26.8 to 263.2)
Walker *et al.* (29); registry study	Astrocytoma, anaplastic	Canada; n=760	-	Rural vs. urban residence, 5-year survival (95% CI): 10.9% (5.9 to 17.5) vs. 18.4% (15.0 to 22.0)
	Glioblastoma	Canada; n=10,950		Rural vs. urban residence, 5-year survival (95% CI): 2.4% (1.8 to 3.2) vs. 3.7% (3.3 to 4.2%)

Study; design	Glioma type/entity	Country; n	Treatment	Survival outcomes
	Oligodendroglioma, anaplastic	Canada; n=730		Rural vs. urban residence, 5-year survival (95% CI): 47.6% (38.9 to 55.9) vs. 35.0% (30.7 to 39.4)
Woo *et al.* (30), retrospective cohort study	Glioblastoma	China (Hong Kong); n=1,010	TMZ	• Median OS (IQR), months: 10.6 (5.2 to 18.4)<br>• 1-year OS: 45%<br>• 2-year OS: 20%
Yuan *et al.* (31); registry study	Astrocytoma, anaplastic	Canada; n=900	-	• 1-year OS (95% CI): 47.9% (44.9 to 50.9)<br>• 2-year OS (95% CI): 33.1% (30.3 to 36.0)<br>• 5-year OS (95% CI): 18.2% (15.8 to 20.7)
	Glioblastoma	Canada; n=14,120		• 1-year OS (95% CI): 26.5% (25.7 to 27.2)<br>• 2-year OS (95% CI): 9.5% (9.0 to 10.0)<br>• 5-year OS (95% CI): 4.0% (3.7 to 4.3)
	Oligodendroglioma	Canada; n=785		• 1-year OS (95% CI): 87.5% (85.8 to 89.0)<br>• 2-year OS (95% CI): 80.7 (78.7 to 82.5)<br>• 5-year OS (95% CI): 65.0 (62.5 to 67.4)
	Oligodendroglioma, anaplastic	Canada; n=580		• 1-year OS (95% CI): 76.5% (73.6 to 79.2)<br>• 2-year OS (95% CI): 61.1% (57.7 to 64.3)<br>• 5-year OS (95% CI): 41.5% (37.9 to 45.0)
Zeng *et al.* (32); retrospective cohort study	Astrocytoma	China; n=44	Surgery	• PFS, 5 years: 61%<br>• PFS, 10 years: 17%<br>• OS, 5 years: 91%<br>• OS, 10 years: 49%
	Oligodendroglioma	China; n=31		• PFS, 5 years: 87%<br>• PFS, 10 years: 42%<br>• OS, 5 years: 100%

Study; design	Glioma type/entity	Country; n	Treatment	Survival outcomes
				• OS, 10 years: 66%
Zentrum für Krebsregisterdaten (33); register study	Astrocytoma, grade 2	Germany	-	Relative 5-year survival, women vs. men: 62% vs. 61%
	Astrocytoma, grade 3			Relative 5-year survival, women vs. men: 33% vs. 34%
	Astrocytoma, grade 4			Relative 5-year survival, women vs. men: 8% vs. 7%

Abbreviations: CI, Confidence Interval; Gy, Gray; IQR, Interquartile Range; PFS, Progression-Free Survival; OS, Overall Survival; PCV, Procarbazine, Lomustine, Vincristine; RCT, Randomized Controlled Trial; RT, Radiotherapy; TMZ, Temozolomide; UK, United Kingdom; US, United States; WHO, World Health Organization.

Note: Data were not selected systematically, but to cover a range of indication and geographic settings. Rows are ordered alphabetically, by author names.

2 Glioma Epidemiology

Table S2 Glioma incidence, prevalence, morbidity, and mortality

Study; design	Glioma type/entity	Country; n	Result
Incidence			
Chen *et al.* (34); registry study	Glioblastoma (elderly patients)	US; n=24,417	ASIR (95% CI), per 100,000 population:<br>• Overall: 13.16 (12.99 to 13.32)<br>• Women: 10.39 (10.20 to 10.59)<br>• Men: 16.85 (16.57 to 17.14)<br>Incidence in the elderly population stable between 2000 and 2017
Dobes *et al.* (35); retrospective cohort study	Glioblastoma	Australia; n=2,275	• ASIR, per 100,000 population: 3.96 (in 2008)<br>• Statistically significant increase (annual percentage change: 2.5 [95% CI:0.4 to 4.6]) since 2000
Dho *et al.* (36); registry study	Astrocytoma, anaplastic	South Korea; n=94	ASIR, per 100,000 person-years:<br>• Overall: 0.15<br>• Men: 0.18

Study; design	Glioma type/entity	Country; n	Result
			• Women: 0.11
	Astrocytoma, diffuse	South Korea; n=3	ASIR, per 100,000 person-years:<br>• Overall: 0.01<br>• Men: 0.00<br>• Women: 0.00
	Astrocytoma, pilocytic	South Korea; n=50	ASIR, per 100,000 person-years:<br>• Overall: 0.17<br>• Men: 0.15<br>• Women: 0.19
	Glioblastoma	South Korea; n=629	ASIR, per 100,000 person-years:<br>• Overall: 0.87<br>• Men: 0.99<br>• Women: 0.78
	Oligodendroglioma	South Korea; n=87	ASIR, per 100,000 person-years:<br>• Overall: 0.14<br>• Men: 0.15<br>• Women: 0.12
	Oligodendroglioma, anaplastic	South Korea; n=59	ASIR, per 100,000 person-years:<br>• Overall: 0.08<br>• Men: 0.08<br>• Women: 0.09
Fuentes-Raspall *et al.* (10); registry study	Astrocytoma, anaplastic	Spain; n=56	ASIR (95% CI), per 100,000 population:<br>• Overall: 0.48 (0.36 to 0.62)<br>• Men: 0.51 (0.34 to 0.75)

Study; design	Glioma type/entity	Country; n	Result
			• Women: 0.44 (0.28 to 0.64)
	Astrocytoma, diffuse	Spain; n=113	ASIR (95% CI), per 100,000 population:<br>• Overall: 0.93 (0.76 to 1.12)<br>• Men: 0.89 (0.66 to 1.19)<br>• Women: 0.99 (0.75 to 1.28)
	Astrocytoma, pilocytic	Spain; n=35	ASIR (95% CI), per 100,000 population:<br>• Overall: 0.28 (0.19 to 0.39)<br>• Men: 0.17 (0.08 to 0.33)<br>• Women: 0.39 (0.25 to 0.59)
	Glioblastoma	Spain; n=463	ASIR (95% CI), per 100,000 population:<br>• Overall: 4.17 (3.80 to 4.57)<br>• Men: 5.05 (4.45 to 5.72)<br>• Women: 3.44 (2.97 to 3.96)
	Oligodendroglioma	Spain; n=21	ASIR (95% CI), per 100,000 population:<br>• Overall: 0.17 (0.10 to 0.26)<br>• Men: 0.12 (0.05 to 0.27)<br>• Women: 0.21 (0.11 to 0.37)
	Oligodendroglioma, anaplastic	Spain; n=8	ASIR (95% CI), per 100,000 population:<br>• Overall: 0.05 (0.02 to 0.11)<br>• Men: 0.09 (0.03 to 0.24)<br>• Women: 0.03 (0.00 to 0.12)
Ji *et al.* (37); registry study	Glioma (including glioblastoma)	Canada; n=32,360	Crude annual incidence, per 100,000 population: 5.45 (ranging, by province, from 1.88 to 6.33)
	Glioblastoma	Canada; n=17,115	Crude annual incidence, per 100,000 population: 2.87 (ranging, by province, from 0.63 to 3.72)

**Study; design**	**Glioma type/entity**	**Country; n**	**Result**
Larjavaara *et al.* (38); retrospective cohort study	Astrocytoma, anaplastic	Finland; n=31	Incidence, per 100,000 population: 0.5
	Astrocytoma, diffuse	Finland; n=46	Incidence, per 100,000 population: 0.7
	Astrocytoma, pilocytic	Finland; n=17	Incidence, per 100,000 population: 0.3
	Glioblastoma	Finland; n=154	Incidence, per 100,000 population: 2.0
	Oligodendroglioma	Finland; n=37	Incidence, per 100,000 population: 0.5
	Glioma	Finland; n=331	ASIR (95% CI), per 100,000 population:<br>• Overall: 4.67 (4.2 to 5.2)<br>• Women: 4.47<br>• Men: 4.90
Ostrom *et al.* (39); registry study	Astrocytoma, anaplastic	US; n=6,537	ASIR (95% CI), per 100,000 population:<br>• Overall: 0.40 (0.39 to 0.41)<br>• Men: 0.46 (0.44 to 0.47)<br>• Women: 0.35 (0.33 to 0.36)
	Astrocytoma, diffuse	US; n=7,793	ASIR (95% CI), per 100,000 population:<br>• Overall: 0.48 (0.47 to 0.49)<br>• Men: 0.55 (0.54 to 0.57)<br>• Women: 0.42 (0.41 to 0.43)
	Astrocytoma, pilocytic	US; n=5,249	ASIR (95% CI), per 100,000 population:<br>• Overall: 0.36 (0.35 to 0.37)<br>• Men: 0.36 (0.35 to 0.37)<br>• Women: 0.35 (0.34 to 0.37)

Study; design	Glioma type/entity	Country; n	Result
	Glioblastoma	US; n=56,421	ASIR (95% CI), per 100,000 population:<br>• Overall: 3.20 (3.17 to 3.23)<br>• Men: 3.99 (3.95 to 4.03)<br>• Women: 2.52 (2.49 to 2.56)
	Oligodendroglioma	US; n=3,753	ASIR (95% CI), per 100,000 population:<br>• Overall: 0.24 (0.23 to 0.25)<br>• Men: 0.28 (0.26 to 0.29)<br>• Women: 0.21 (0.20 to 0.22)
	Oligodendroglioma, anaplastic	US; n=1,718	ASIR (95% CI), per 100,000 population:<br>• Overall: 0.11 (0.10 to 0.11)<br>• Men: 0.12 (0.11 to 0.13)<br>• Women: 0.09 (0.09 to 0.10)
	Glioma, malignant, not otherwise specified	US; n=7,238	ASIR (95% CI), per 100,000 population:<br>• Overall: 0.46 (0.45 to 0.47)<br>• Men: 0.49 (0.48 to 0.51)<br>• Women: 0.44 (0.42 to 0.45)
Pouchieu *et al.* (40); registry study	Astrocytoma, anaplastic	France; n=56	ASIR, per 100,000 population:<br>• Overall: 0.31<br>• Men: 0.33<br>• Women: 0.30
	Astrocytoma, diffuse	France; n=42	ASIR, per 100,000 population:<br>• Overall: 0.23<br>• Men: 0.30<br>• Women: 0.17

**Study; design**	**Glioma type/entity**	**Country; n**	**Result**
	Astrocytoma, pilocytic	France; n=34	ASIR, per 100,000 population:<br>• Overall: 0.19<br>• Men: 0.20<br>• Women: 0.19
	Glioblastoma	France; n=945	ASIR, per 100,000 population:<br>• Overall: 5.3<br>• Men: 6.1<br>• Women: 4.2
	Oligodendroglioma	France; n=28	ASIR, per 100,000 population:<br>• Overall: 0.15<br>• Men: 0.16<br>• Women: 0.14
	Oligodendroglioma, anaplastic	France; n=13	ASIR, per 100,000 population:<br>• Overall: 0.07<br>• Men: 0.05<br>• Women: 0.09
Sehmer *et al.* (41); retrospective cohort study	Glioma	UK; n=435	ASIR (95% CI), per 100,000 population: 7.1 (6.5 to 7.8)
Wanis *et al.* (42); registry study	Glioblastoma	UK; n=133,669 (overall)	• ASIR, per 100,000 population, men, in 2017: 7.34<br>• ASIR, per 100,000 population, women, in 2017: 4.45<br>• Increases in ASIRs for both sexes over time (values were 3.27 and 2.00 in 1995, respectively)<br>• Suggested explanation for the increase: improved clinical practice and cancer registration

Study; design	Glioma type/entity	Country; n	Result
Mortality			
Ostrom *et al*. (39); registry study	Glioma	US; n=	Age-adjusted mortality rate, per 100,000 population: 4.33
Histology distribution			
Larjavaara *et al*. (38); retrospective cohort study	-	Finland; n=331	Of all gliomas:<br>• Glioblastoma: 47.0%<br>• Astrocytoma, diffuse: 14%<br>• Oligodendroglioma: 11%<br>• Astrocytoma, anaplastic: 9.4%<br>• Astrocytoma, pilocytic: 5.1%<br>• Astrocytoma, other: 0.6%<br>• Gliomas, mixed: 11.0%<br>• Ependymomas: 3.0%
Ostrom *et al*. (39); registry study	-	US; n=100,619	Of primary brain and other CNS gliomas:<br>• Glioblastoma: 56.1%<br>• Astrocytoma, diffuse: 7.7%<br>• Glioma, malignant, not otherwise specified: 7.2%<br>• Ependymal tumors: 6.8%<br>• Astrocytoma, anaplastic: 6.5%<br>• Oligodendroglioma: 5.4%<br>• Astrocytoma, pilocytic: 5.2%<br>• Oligoastrocytic tumors: 3.0%<br>• Gliomas, other: 2.1%

Study; design	Glioma type/entity	Country; n	Result
Zentrum für Krebsregisterdaten (33); registry study	-	Germany	Of malignant brain tumors:<br>• Glioblastoma: 67.8% in women, 69.3% in men<br>• Astrocytoma, grade 3: 8.9% in women, 8.5% in men<br>• Astrocytoma, grade 2: 8.7% in women, 8.1% in men<br>• Other: 13.9% in women, 11.7% in men

Abbreviations: ASIR, Age-Standardized Incidence Rate; CNS, Central Nervous System; UK, United Kingdom; US, United States.

Note: Unless specified differently, all incidence values, including for ASIRs, are expressed per 100,000 population

3 References

1. Blakstad H, Brekke J, Rahman MA, Arnesen VS, Miletic H, Brandal P, et al. Survival in a consecutive series of 467 glioblastoma patients: association with prognostic factors and treatment at recurrence at two independent institutions. *PLoS One* (2023) 18:e0281166. doi: 10.1371/journal.pone.0281166

2. Brown NF, Ottaviani D, Tazare J, Gregson J, Kitchen N, Brandner S, et al. Survival outcomes and prognostic factors in glioblastoma. *Cancers* (2022) 14:3161. doi: 10.3390/cancers14133161

3. Cairncross JG, Wang M, Shaw E, Jenkins R, Brachman D, Buckner J, et al. Phase III trial of chemoradiotherapy for anaplastic oligodendroglioma: long-term results of RTOG 9402. *J Clin Oncol* (2013) 31:337–343. doi: 10.1200/JCO.2012.43.2674

4. Cheo STT, Lim GH, Lim KHC. Glioblastoma multiforme outcomes of 107 patients treated in two Singapore institutions. *Singapore Med J* (2017) 58:41–45. doi: 10.11622/smedj.2016044

5. Claus EB, Walsh KM, Wiencke J, Molinaro AM, Wiemels JL, Schildkraut JM, et al. Survival and low grade glioma: the emergence of genetic information. *Neurosurg Focus* (2015) 38:E6. doi: 10.3171/2014.10.FOCUS12367

6. Decavèle M, Gatulle N, Weiss N, Rivals I, Idbaih A, Demeret S, et al. One-year survival of patients with high-grade glioma discharged alive from the intensive care unit. *J Neurol* (2021) 268:516–525. doi: 10.1007/s00415-020-10191-0

7. De Witt Hamer PC, Ho VKY, Zwinderman AH, Ackermans L, Ardon H, Boomstra S, et al. Between-hospital variation in mortality and survival after glioblastoma surgery in the Dutch Quality Registry for Neuro Surgery. *J Neurooncol* (2019) 144:313–323. doi: 10.1007/s11060-019-03229-5

8. Efremov L, Abera SF, Bedir A, Vordermark D, Medenwald D. Patterns of glioblastoma treatment and survival over a 16-years period: pooled data from the German Cancer Registries. *J Cancer Res Clin Oncol* (2021) 147:3381–3390. doi: 10.1007/s00432-021-03596-5

9. Fabbro-Peray P, Zouaoui S, Darlix A, Fabbro M, Pallud J, Rigau V, et al. Association of patterns of care, prognostic factors, and use of radiotherapy-temozolomide therapy with survival in patients with newly diagnosed glioblastoma: a French national population-based study. *J Neurooncol* (2019) 142:91–101. doi: 10.1007/s11060-018-03065-z

10. Fuentes-Raspall R, Solans M, Roca-Barceló A, Vilardell L, Puigdemont M, Del Barco S, et al. Descriptive epidemiology of primary malignant and non-malignant central nervous tumors in Spain: results from the Girona Cancer Registry (1994-2013). *Cancer Epidemiol* (2017) 50:1–8. doi: 10.1016/j.canep.2017.07.005

11. Girardi F, Matz M, Stiller C, You H, Marcos Gragera R, Valkov MY, et al. Global survival trends for brain tumors, by histology: analysis of individual records for 556,237 adults

diagnosed in 59 countries during 2000-2014 (CONCORD-3). *Neuro-Oncol* (2023) 25:580–592. doi: 10.1093/neuonc/noac217

12. Gramatzki D, Dehler S, Rushing EJ, Zaugg K, Hofer S, Yonekawa Y, et al. Glioblastoma in the Canton of Zurich, Switzerland revisited: 2005 to 2009. *Cancer* (2016) 122:2206–2215. doi: 10.1002/cncr.30023

13. Gramatzki D, Roth P, Rushing EJ, Weller J, Andratschke N, Hofer S, et al. Bevacizumab may improve quality of life, but not overall survival in glioblastoma: an epidemiological study. *Ann Oncol* (2018) 29:1431–1436. doi: 10.1093/annonc/mdy106

14. Hansen S, Rasmussen BK, Laursen RJ, Kosteljanetz M, Schultz H, Nørgård BM, et al. Treatment and survival of glioblastoma patients in Denmark: the Danish Neuro-Oncology Registry 2009-2014. *J Neurooncol* (2018) 139:479–489. doi: 10.1007/s11060-018-2892-7

15. Hertler C, Felsberg J, Gramatzki D, Le Rhun E, Clarke J, Soffietti R, et al. Long-term survival with IDH wildtype glioblastoma: first results from the ETERNITY Brain Tumor Funders' Collaborative Consortium (EORTC 1419). *Eur J Cancer* (2023) 189:112913. doi: 10.1016/j.ejca.2023.05.002

16. Marra JS, Mendes GP, Yoshinari GH, da Silva Guimarães F, Mazin SC, de Oliveira HF. Survival after radiation therapy for high-grade glioma. *Rep Pract Oncol Radiother* (2019) 24:35–40. doi: 10.1016/j.rpor.2018.09.003

17. Mathiesen T, Peredo I, Lönn S. Two-year survival of low-grade and high-grade glioma patients using data from the Swedish Cancer Registry. *Acta Neurochir (Wien)* (2011) 153:467–471. doi: 10.1007/s00701-010-0894-0

18. Narita Y, Shibui S, Committee of Brain Tumor Registry of Japan. Trends and outcomes in the treatment of gliomas based on data during 2001-2004 from the Brain Tumor Registry of Japan. *Neurol Med Chir (Tokyo)* (2015) 55:286–295. doi: 10.2176/nmc.ra.2014-0348

19. Ostrom QT, Shoaf ML, Cioffi G, Waite K, Kruchko C, Wen PY, et al. National-level overall survival patterns for molecularly-defined diffuse glioma types in the United States. *Neuro-Oncol* (2023) 25:799–807. doi: 10.1093/neuonc/noac198

20. Picart T, Meyronet D, Pallud J, Dumot C, Metellus P, Zouaoui S, et al. Management, functional outcomes and survival in a French multicentric series of 118 adult patients with cerebellar glioblastoma. *J Cancer Res Clin Oncol* (2021) 147:1843–1856. doi: 10.1007/s00432-020-03474-6

21. Reiner AS, Lobaugh SM, Gonen S, Diamond EL, Panageas KS. A population-based study of treatment and survival in older glioma patients. *JNCI Cancer Spectr* (2022) 6:pkac010. doi: 10.1093/jncics/pkac010

22. Shieh L-T, Ho C-H, Guo H-R, Huang C-C, Ho Y-C, Ho S-Y. Epidemiologic features, survival, and prognostic factors among patients with different histologic variants of glioblastoma: analysis of a nationwide database. *Front Neurol* (2021) 12:659921. doi: 10.3389/fneur.2021.659921

23. Solomons MR, Jaunmuktane Z, Weil RS, El-Hassan T, Brandner S, Rees JH. Seizure outcomes and survival in adult low-grade glioma over 11 years: living longer and better. *Neuro-Oncol Pract* (2020) 7:196–201. doi: 10.1093/nop/npz056

24. Tabei Y, Kobayashi K, Saito K, Shimizu S, Suzuki K, Sasaki N, et al. Survival in patients with glioblastoma at a first progression does not correlate with isocitrate dehydrogenase (IDH)1 gene mutation status. *Jpn J Clin Oncol* (2021) 51:45–53. doi: 10.1093/jjco/hyaa162

25. Tesileanu CMS, Dirven L, Wijnenga MMJ, Koekkoek JAF, Vincent AJPE, Dubbink HJ, et al. Survival of diffuse astrocytic glioma, IDH1/2 wildtype, with molecular features of glioblastoma, WHO grade IV: a confirmation of the cIMPACT-NOW criteria. *Neuro-Oncol* (2020) 22:515–523. doi: 10.1093/neuonc/noz200

26. van den Bent MJ, Tesileanu CMS, Wick W, Sanson M, Brandes AA, Clement PM, et al. Adjuvant and concurrent temozolomide for 1p/19q non-co-deleted anaplastic glioma (CATNON; EORTC study 26053-22054): second interim analysis of a randomised, open-label, phase 3 study. *Lancet Oncol* (2021) 22:813–823. doi: 10.1016/S1470-2045(21)00090-5

27. Vargas López AJ, Fernández Carballal C, Valera Melé M, Rodríguez-Boto G. Survival analysis in high-grade glioma: the role of salvage surgery. *Neurologia* (2023) 38:21–28. doi: 10.1016/j.nrleng.2020.04.032

28. Villani V, Casini B, Tanzilli A, Lecce M, Rasile F, Telera S, et al. The Glioma-IRE project - molecular profiling in patients with glioma: steps toward an individualized diagnostic and therapeutic approach. *J Transl Med* (2023) 21:215. doi: 10.1186/s12967-023-04057-y

29. Walker EV, Ross J, Yuan Y, Smith TR, Davis FG. Brain cancer survival in Canada 1996-2008: effects of sociodemographic characteristics. *Curr Oncol* (2019) 26:e292–e299. doi: 10.3747/co.26.4273

30. Woo PYM, Yau S, Lam T-C, Pu JKS, Li L-F, Lui LCY, et al. Patterns of care and survival of Chinese glioblastoma patients in the temozolomide era: a Hong Kong population-level analysis over a 14-year period. *Neuro-Oncol Pract* (2023) 10:50–61. doi: 10.1093/nop/npac069

31. Yuan Y, Shi Q, Li M, Nagamuthu C, Andres E, Davis FG. Canadian brain cancer survival rates by tumour type and region: 1992–2008. *Can J Public Health Rev Can Santé Publique* (2016) 107:e37–e42. doi: 10.17269/cjph.107.5209

32. Zeng L, Mei Q, Li H, Ke C, Yu J, Chen J. A survival analysis of surgically treated incidental low-grade glioma patients. *Sci Rep* (2021) 11:8522. doi: 10.1038/s41598-021-88023-y

33. Zentrum für Krebsregisterdaten. Zentrales Nervensystem. Berlin: Robert-Koch-Institut (2021). https://www.krebsdaten.de/Krebs/DE/Content/Publikationen/Krebs_in_Deutschland/kid_2021/kid_2021_c70_c72_zns.pdf?__blob=publicationFile [Accessed August 24, 2023]

34. Chen B, Chen C, Zhang Y, Xu J. Recent incidence trend of elderly patients with glioblastoma in the United States, 2000–2017. *BMC Cancer* (2021) 21:54. doi: 10.1186/s12885-020-07778-1

35. Dobes M, Khurana VG, Shadbolt B, Jain S, Smith SF, Smee R, et al. Increasing incidence of glioblastoma multiforme and meningioma, and decreasing incidence of Schwannoma (2000-

2008): findings of a multicenter Australian study. *Surg Neurol Int* (2011) 2:176. doi: 10.4103/2152-7806.90696

36. Dho Y-S, Jung K-W, Ha J, Seo Y, Park C-K, Won Y-J, et al. An updated nationwide epidemiology of primary brain tumors in Republic of Korea, 2013. *Brain Tumor Res Treat* (2017) 5:16–23. doi: 10.14791/btrt.2017.5.1.16

37. Ji X, Alakel A, Ghazawi FM, Tsang M, Zubarev A, Lasry OJ, et al. Investigation of incidence and geographic distribution of gliomas in Canada from 1992 to 2010: a national population-based study highlighting the importance of exposure to airport operations. *Front Oncol* (2023) 13:1190366. doi: 10.3389/fonc.2023.1190366

38. Larjavaara S, Mäntylä R, Salminen T, Haapasalo H, Raitanen J, Jääskeläinen J, et al. Incidence of gliomas by anatomic location. *Neuro-Oncol* (2007) 9:319–325. doi: 10.1215/15228517-2007-016

39. Ostrom QT, Price M, Neff C, Cioffi G, Waite KA, Kruchko C, et al. CBTRUS statistical report: primary brain and other central nervous system tumors diagnosed in the United States in 2016-2020. *Neuro-Oncol* (2023) 25:iv1–iv99. doi: 10.1093/neuonc/noad149

40. Pouchieu C, Gruber A, Berteaud E, Ménégon P, Monteil P, Huchet A, et al. Increasing incidence of central nervous system (CNS) tumors (2000-2012): findings from a population based registry in Gironde (France). *BMC Cancer* (2018) 18:653. doi: 10.1186/s12885-018-4545-9

41. Sehmer EAJ, Hall GJ, Greenberg DC, O'Hara C, Wallingford SC, Wright KA, et al. Incidence of glioma in a northwestern region of England, 2006-2010. *Neuro-Oncol* (2014) 16:971–974. doi: 10.1093/neuonc/not301

42. Wanis HA, Møller H, Ashkan K, Davies EA. The incidence of major subtypes of primary brain tumors in adults in England 1995-2017. *Neuro-Oncol* (2021) 23:1371–1382. doi: 10.1093/neuonc/noab076
